# Extracellular vesicle miR-93-5p cargo regulates glomerular endothelial cell damage in Alport syndrome

**DOI:** 10.1172/jci.insight.197643

**Published:** 2026-03-23

**Authors:** Charmi Dedhia, Valentina Villani, Xiaogang Hou, Paolo Neviani, Geremy Clair, Mohammadreza Kasravi, Cristina Grange, Paolo Cravedi, Paola Aguiari, Velia Alcala, Giuseppe Orlando, Xue-Ying Song, Jonathan E. Zuckerman, Roger E. De Filippo, Stefano Da Sacco, Sargis Sedrakyan, Benedetta Bussolati, Laura Perin

**Affiliations:** 1 The GOFARR Laboratory, The Saban Research Institute, Division of Urology, and; 2 Extracellular Vesicle Core, The Saban Research Institute, Children’s Hospital Los Angeles, Los Angeles, California, USA.; 3 Biological Sciences Division, Pacific Northwest National Laboratory, Richland, Washington, USA.; 4 Department of Medical Sciences, University of Turin, Turin, Italy.; 5 Department of Medicine, Translational Transplant Research Center, Icahn School of Medicine at Mount Sinai, New York, New York, USA.; 6 Section of Transplantation, Department of Surgery, Wake Forest University School of Medicine, Winston Salem, North Carolina, USA.; 7 Applied Genomics, Computation, and Translational Core, Cedars-Sinai Medical Center Los Angeles, California, USA.; 8 Department of Pathology and Lab Medicine, David Geffen School of Medicine at UCLA, Los Angeles, California, USA.; 9 Department of Urology, Keck School of Medicine, University of Southern California, Los Angeles, California, USA.

**Keywords:** Cell biology, Nephrology, Chronic kidney disease

## Abstract

Modulation of miRNA expression in glomerular cells is associated with renal disease. Here, we investigated the role of miR-93-5p in mitigating glomerular damage in Alport syndrome and whether the disease-modifying activity of extracellular vesicles from human amniotic fluid stem cells (hAFSC-EVs) is mediated by their miR-93-5p cargo. We identified downregulation of miR-93-5p specifically in glomerular endothelial cells in Alport syndrome along disease progression. Silencing of miR-93-5p in hAFSC-EVs changed the transcriptomic and proteomic profile, regulating EV disease-modifying activity. Compared with naive hAFSC-EVs, silenced hAFSC-EVs did not rescue glomerular endothelial function in vitro and did not restore kidney function in vivo. We established that hAFSC-EVs regulate VEGFR1 and VEGFR2 signaling by miR-93-5p cargo transfer, highlighting that miR-93-5p can restore glomerular endothelial cell biology. Spatial transcriptomics analysis of hAFSC-EV–injected kidneys showed that these EVs can reverse pathways altered during disease progression by stimulating proregenerative processes, specifically in the glomerulus, by regulating miR-93-5p targets. Alteration of glomerular endothelial cell transcriptomics and miR-93-5p targets was also confirmed in biopsies of patients with Alport syndrome using spatial molecular imaging. We demonstrated the critical role of miR-93-5p in glomerular endothelial cells and the capability of hAFSC-EVs to regulate miR-93-5p and its targets in Alport syndrome.

## Abstract 2

miR-93-5p-specific human AFSC-EVs cargo restores glomerular endothelial function in Alport syndrome by modulating VEGFR signaling and by promoting glomerular repair.

## Graphical abstract

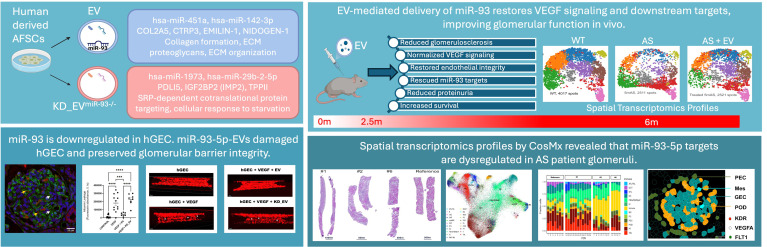

## Introduction

Disturbance of the crosstalk between glomerular cells and changes in their interaction with the glomerular basement membrane (GBM) activate disease processes that lead to kidney failure (1). Alport syndrome (AS) is a progressive glomerular disease caused by mutations in the *COL4A3*, *COL4A4*, and *COL4A5* genes (2, 3), resulting in the improper assembly of the GBM and leading to changes in cell-matrix interactions and altered glomerular cell crosstalk (4–6). Patients with AS, in addition to kidney disease, also present with auditory and ocular symptoms (2, 7, 8). AS management is centered on angiotensin-converting enzyme inhibitors or angiotensin II receptor blockers, and recently on drugs like sparsentan (dual endothelin and angiotensin II receptor antagonist) or sodium-glucose cotransporter 2 inhibitors, which show stronger effects in preserving kidney function (9, 10). Even though these therapies delay the progression to end-stage renal disease, there is an unmet need for new disease-modifying therapies that can directly repair or stabilize glomerular cell biology.

AS is considered a podocyte-centric disease, but we (11–13) and others (14) showed a central role of glomerular endothelial cells (GECs) in AS as well as in other renal diseases. The crosstalk between podocytes and GECs is essential for the function of the filtration barrier (1, 15). For example, GECs are particularly vulnerable to changes in VEGF signaling produced by podocytes (1, 15, 16), as VEGF is a crucial regulator of glomerular capillary homeostasis (17) and it is implicated not only in AS (18) but also in various renal pathologies (1, 16).

Extracellular vesicles (EVs) are fundamental modulators of cell-cell communication (19, 20) and, in particular, stem cell–derived EVs play an important role in modulating molecular pathways ranging from fibrosis to immunomodulation to tissue repair, thus slowing down disease progression in many conditions, including renal disease (21–23). Although their organ-protective role is recognized, the mechanisms involved in the EV-mediated glomerular protection are poorly understood, and further investigation into how EVs function as regulators of cell-signaling dynamics is essential to elucidate their contribution to renal protection.

We previously demonstrated (11) that EVs derived from mouse amniotic fluid stem cells (AFSC-EVs) can modulate VEGF signaling in the glomerulus in AS (*Col4a5^–/–^*) mice, characterized by a mutation in the α5 chain of collagen IV, the most prominent form of AS in humans (2, 3).

While previous studies using mouse-derived EVs have demonstrated renoprotective effects in experimental models, key questions remain regarding the therapeutic potential of clinically relevant human EV platforms. Therefore, to facilitate the EV clinical translation for chronic kidney disease (CKD), we here studied and characterized human-derived EVs from amniotic fluid.

Among the various components of the EV cargo (proteins, nucleic acids, lipids), microRNAs (miRs), small noncoding RNA molecules (24), are considered key players in exerting EV function; once delivered to the target cells, they modulate gene expression, influencing cell proliferation, differentiation, apoptosis, and other biological processes (25–27).

We previously reported the first evidence that miR-93-5p (hereafter referred to as miR-93), a potent regulator of VEGF signaling (28), plays a key role in GEC biology during AS progression and that miR-93–specific EV cargo transfer is essential for restoring GEC function in AS. Our findings provide insights into the mechanistic role of EVs in regulating GEC biology and emphasize the potential translation of human AFSC-EVs to the clinic as a treatment for patients with AS and other forms of glomerulopathy.

## Results

### miR-93 expression changes in glomerular cells during AS disease progression.

**Figure 1:**
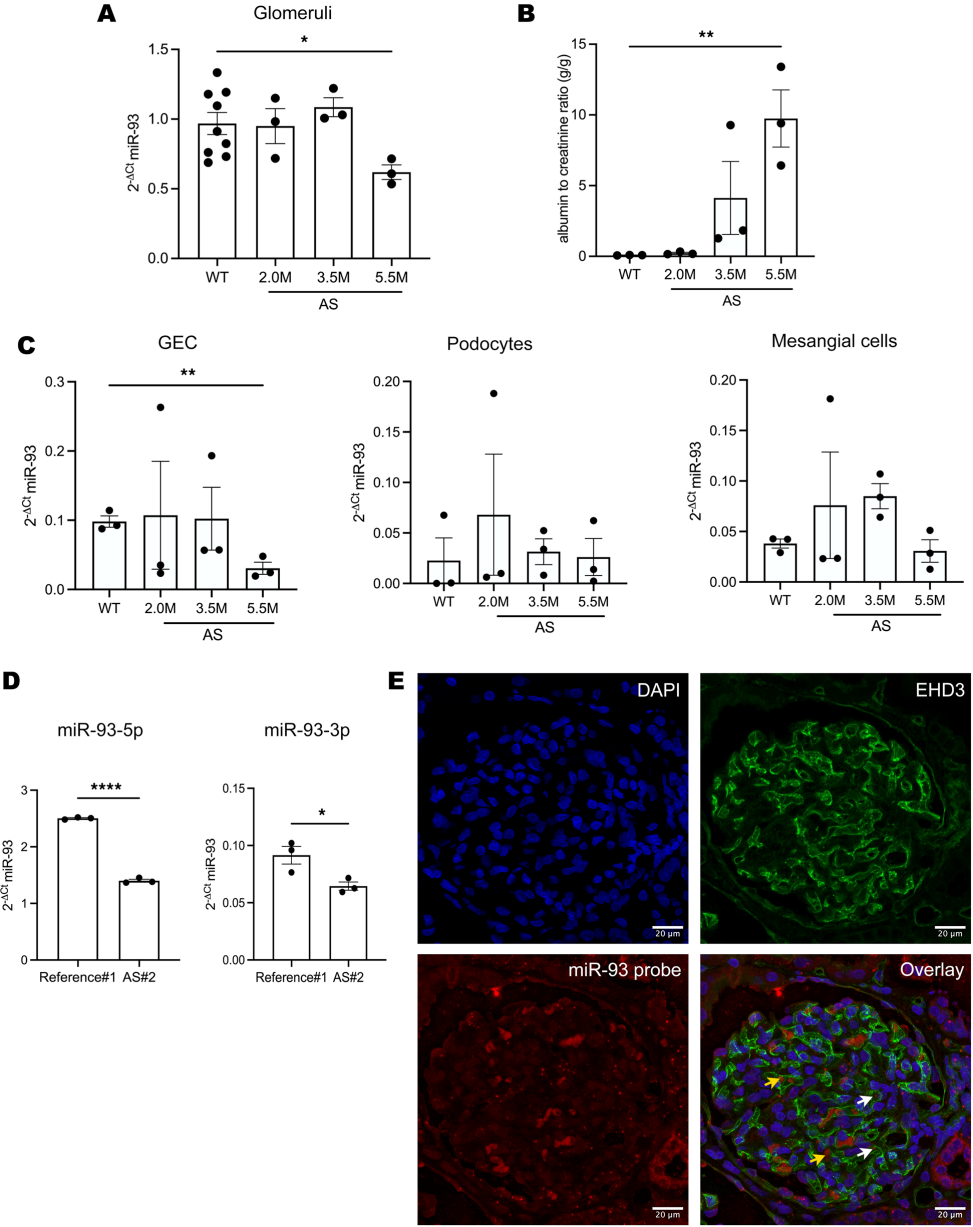
miR-93 expression decreases along disease progression in mice and humans. (**A**) Graph showing miR-93 expression in glomeruli isolated from WT mice (C57BL/6J) at 4 months old (4m) and AS (*Col4a5^–/–^*; B6.*Cg-Col4a5^tm1Yseg^*/J) mice at 2m, 3.5m, and 5.5m. (**B**) Graph showing proteinuria levels, measured as albumin-to-creatinine ratio in WT mice and AS mice at 2m, 3.5m, and 5.5m. (**C**) Graph showing miR-93 expression in GECs, podocytes, and mesangial cells isolated from WT (4m), and AS mice at 2m, 3.5m, and 5.5m. No significant difference in miR-93 expression was noted in podocytes and mesangial cells. (**D**) Graph showing miR-93-5p (left) and miR-93-3p (right) expression in the kidney cortex of a human biopsy of AS patient (no. 2; male, 17 years old) compared to healthy tissue of a partial nephrectomy (used as reference; reference no. 1; male, 66 years old), showing that miR-93-5p is less expressed in AS and that miR-93-3p is much less expressed compared with miR-93-5p. (**E**) Representative images of human glomeruli, healthy tissue (reference no. 3) of a partial nephrectomy (male, 7 years old) showing miR-93 expression by in situ hybridization. Upper left corner: DAPI in blue. Upper right corner: EHD3 (green), identifying GECs. Lower left corner: miR-93 probe (red). Lower right corner: merged panels. White arrows: GECs showing coexpression of the miR-93 probe and EHD3. Yellow arrows: RBCs (autofluorescence in red). Scale bars: 20 μm. For miR-93 expression, small nuclear RNA U6 was used as a reference to calculate relative expression. All experiments were run in triplicate. All values are reported as mean ± SEM. Statistical significance was assessed using 1-way ANOVA with post hoc uncorrected Fisher’s LSD test. *P < 0.05; **P < 0.01; ****P < 0.0001.

To study the role of miR-93 in AS, we first determined the expression of miR-93 within the glomeruli of AS versus WT mice along disease progression, showing that miR-93 is downregulated in glomeruli in AS mice at 5.5 months of age (5.5mAS mice) (Figure 1A), when the level of proteinuria is high (Figure 1B). We detected a significant downregulation of miR-93 expression specifically in GECs in AS, but not in podocytes or mesangial cells (Figure 1C and Supplemental Figure 1; supplemental material available online with this article; https://doi.org/10.1172/jci.insight.197643DS1). miR-93 exists in 2 mature isoforms (miR-93-5p and miR-93-3p); the 3p form is not highly expressed in WT cells and does not change along disease progression in AS cells (Supplemental Figure 2, A–C); therefore, our study focused on the 5p form.

We confirmed that miR-93 expression is downregulated in biopsies of patients with AS and confirmed the biological relevance of the 5p form versus the 3p isoform also in human samples (Figure 1D). Since the mouse data showed that miR-93 is expressed in GECs, we confirmed by in situ hybridization that miR-93 is mainly expressed in GECs also in human glomeruli (Figure 1E and Supplemental Figure 2D). To characterize the glomerular gene signature in AS and to determine whether miR-93 targets are altered in AS glomeruli, we performed bulk RNA-seq analysis on glomeruli isolated from AS mice and age-matched WT mice (Supplemental Figure 3, Supplemental Dataset 1, and NCBI Gene Expression Omnibus [GEO] dataset GSE318476). Principal component analysis (PCA) revealed a clear separation between AS and WT glomeruli (Supplemental Figure 3A), and transcriptional differences were also confirmed by hierarchical clustering (Supplemental Figure 3B). A volcano plot (Supplemental Figure 3C) revealed a significant shift in gene expression in AS versus WT. Specifically, *Tnfrsf21* and *Tgfbr2*, predicted miR-93 targets, were altered in AS glomeruli (Supplemental Figure 3D), thus correlating transcriptional changes in AS with changes in miR-93 targets. Our studies were performed in male AS mice because of the X-linked nature of the disease, and even though heterozygous females were not the focus of our analysis, alteration of miR-93 targets was also present in glomeruli of female AS mice compared with age-matched WT female mice (Supplemental Figure 4, A–D, and Supplemental Dataset 1). Importantly, even if heterozygous females were to present with milder disease with no evident changes in proteinuria during our analysis timeline (Supplemental Figure 4E), they were analyzed to document that their glomerular transcriptome is different versus WT and that miR-93 targets are also modified in the milder form of AS disease.

### Loss of miR-93 alters the EV cargo.

**Figure 2:**
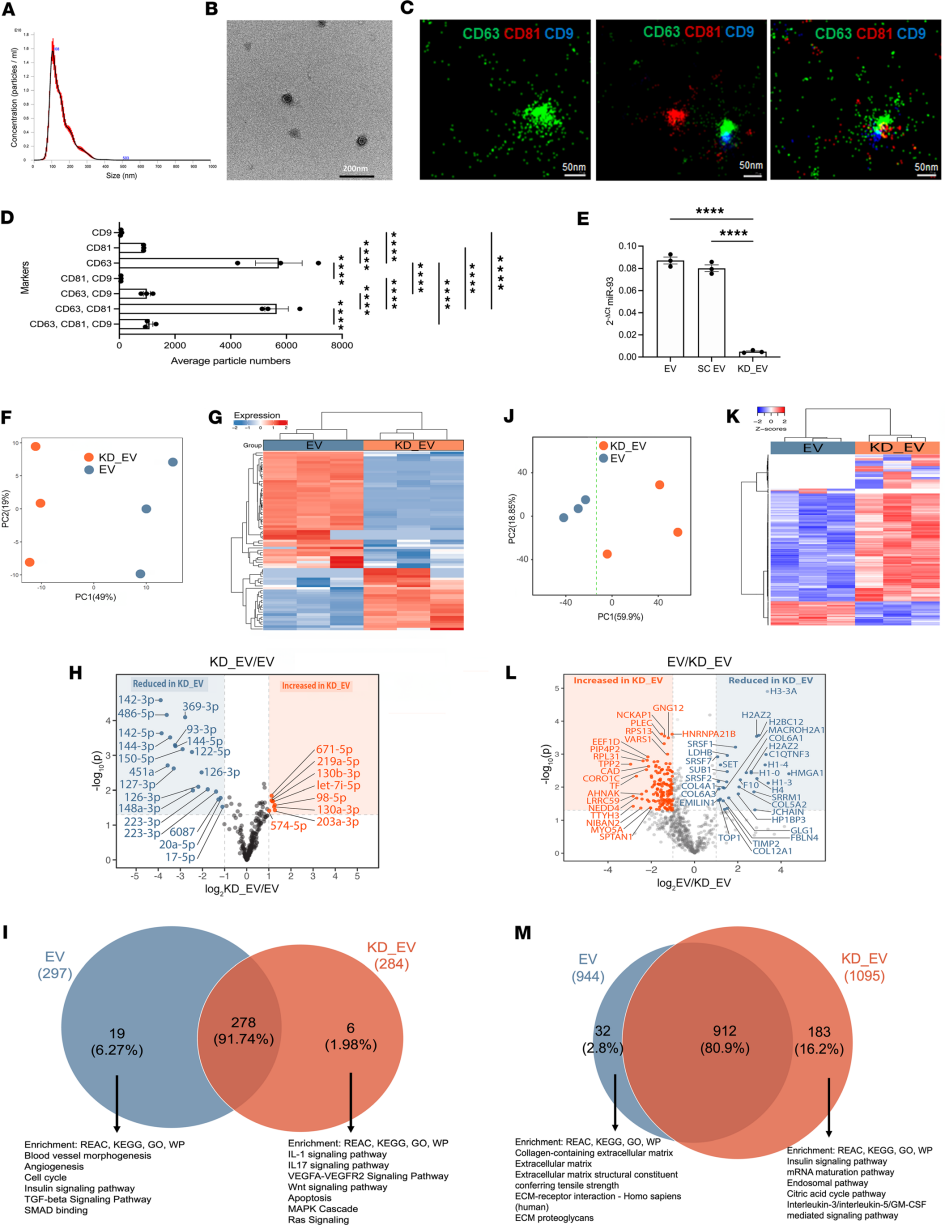
Loss of miR-93 alters EV cargo. (**A**) Nanoparticle tracking analysis representing the average mode of hAFSC-EVs (~108 nm). (**B**) TEM representing typical EV morphology (scale bar: 200 nm). (**C** and **D**) Super-resolution microscopy of the surface tetraspanins CD9, CD63, and CD81 (**C**), with a graph representing the single-, double-, and triple-positive fractions (**D**). Scale bars: 50 nm. (**E**) miR-93 expression in EVs derived from hAFSCs and KD hAFSCs. SC EVs: EVs treated with scramble control for miR-93. (**F**) Principal component analysis (PCA) of the miR sequencing of EVs (blue, *n* = 3) and KD-EVs (orange, *n* = 3) showing the distribution of samples along PC1 (49% variance) and PC2 (19% variance). (**G**) Hierarchical clustering heatmap of miRs expression in EVs (green, left) and KD_EVs (orange, right), showing a shift following miR-93 KD. (**H**) Volcano plot showing the differentially expressed (DE) miRs, upregulated (red) or downregulated (blue), in EVs versus KD_EVs (FC > 1.5 or FC < −1.5; *P* < 0.05). (**I**) Venn diagram representing DE miRs in EVs (blue, left) and KD_EVs (orange, right). Enriched pathways are shown below arrows. (**J**) PCA from the proteomic analysis of EVs (blue, *n* = 3) and KD_EVs (orange, *n* = 3) showing separation along PC1 (59.9% variance) and PC2 (18.85% variance). (**K**) Hierarchical clustering representing expression of proteins in EVs (green group, right) and KD_EVs (orange, left), showing shifts following KD_EVs. (**L**) Volcano plot of DE proteins in KD_EVs vs. EVs (log_2_FC > 0 or log_2_FC < 0; adjusted *P* < 0.05) upregulated (red) and downregulated (blue). (**M**) Venn diagram of DE proteins in EVs (blue, left) and KD_EVs (orange, right). The enriched pathways of 32 EV and 183 KD_EV proteins are indicated. miR-93 expression was normalized to small nuclear RNA U6 and measured in triplicate. Data are reported as mean ± SEM. ****P < 0.0001 by 1-way ANOVA with post hoc uncorrected Fisher’s LSD test (**D** and **E**).

miR-93 is one of the most highly expressed miRs in human EVs (Supplemental Dataset 2), with comparable expression in mouse and human EVs (Supplemental Figure 5A). For data reproducibility, we have characterized (and used in all experiments) EVs derived from our established human AFSC (hAFSC) clonal line, as published previously (11, 29–31). The data indicate that in 24 hours, 1 × 10^6^ hAFSCs produced 2.8 × 10^10^ EVs, with a mode size of approximately 108 nm (Figure 2A) and had an intact membrane, as confirmed by TEM (Figure 2B). Super-resolution microscopy confirmed the presence of the tetraspanins CD9, CD63, and CD81, known to be commonly expressed in EVs (19). The majority of EVs were CD63^+^ or CD63^+^CD81^+^, with smaller fractions of CD81^+^, CD63^+^CD9^+^, and CD63^+^CD81^+^CD9^+^ (Figure 2, C and D). We also determine that hAFSC-EVs are CD63^+^VEGFR2^+^, CD63^+^CD81^+^VEGFR2^+^, CD81^+^VEGFR1^+^, CD63^+^VEGFR1^+^, and CD81^+^CD62^+^VEGFR1^+^ (Supplemental Figure 5, B–E).

To understand how miR-93 expression affects EV cargo, we generated miR-93–knockdown EVs (hAFSC-EVs*^miR-93–/–^*, hereafter KD_EVs) using a transient antagomiR method and confirmed silencing in the cells (Supplemental Figure 5F) and EVs (Figure 2E). We performed miR sequencing on naive EVs and KD_EVs (Supplemental Dataset 2). Analysis showed a clear separation between KD_EVs and EVs (Figure 2F), and an overall shift in miR expression between the 2 groups (Figure 2G). A volcano plot indicates that miR-93 KD resulted in significant changes in specific miRs (Figure 2H); the most significantly upregulated and downregulated miRs in KD_EVs are shown. KD of miR-93 resulted in upregulation of miR-1973 and miR-29b-2-5p (only in KD_EVs) and downregulation of miR-4325p, miR-151a-3p, and miR-10b-5p expression in the KD_EVs (Supplemental Dataset 2). Prediction targets of the above-cited miRs include genes that play a key role in CKD progression, like *PDGRB*, *WNK1*, *TGFb1*, *VEGF*, *FBN1*, *FN*, and *CADM2* (32–39), with miR-432-5p having the highest predicted target gene, *COL4A5*. We identified (Figure 2I) the exclusively upregulated miRs in EVs (19 miRs) and KD_EVs (6 miRs). Enrichment analysis of these miRs’ predicted genes in EVs is associated with pathways related to blood vessel morphogenesis, angiogenesis, cell cycle, and the insulin signaling pathway, whereas the analysis of the miR predicted genes in KD_EVs identified pathways such as IL-1 signaling, IL-17 signaling, VEGF and WNT signaling, and apoptosis.

We performed proteomics on the same EVs (Supplemental Dataset 3). Analysis showed that KD_EVs differ from the naive EVs (Figure 2J); protein cargo changes are shown in the hierarchical clustering (Figure 2K) and in the volcano plot (Figure 2L, the most upregulated and downregulated proteins in KD_EVs are shown). From the total identified proteins, 183 were uniquely present in KD_EVs and 32 in the naive EVs (Figure 2M). These uniquely expressed proteins in the KD_EVs are involved in pathways like the insulin signaling pathway, mRNA maturation pathway, and endosomal pathway. Proteins exclusively upregulated in EVs instead displayed enrichment for pathways related to collagen-containing ECM, ECM structural constituents, and cell-matrix adhesion pathways, all important pathways altered during AS progression (Figure 2M). The results from the miR sequencing and the proteomics show that KD of miR-93 expression induces changes in miRs and protein content that are relevant and known to contribute to AS disease.

### EVs regulate the miR-93/VEGFR1/VEGFR2 axis in GECs.

**Figure 3:**
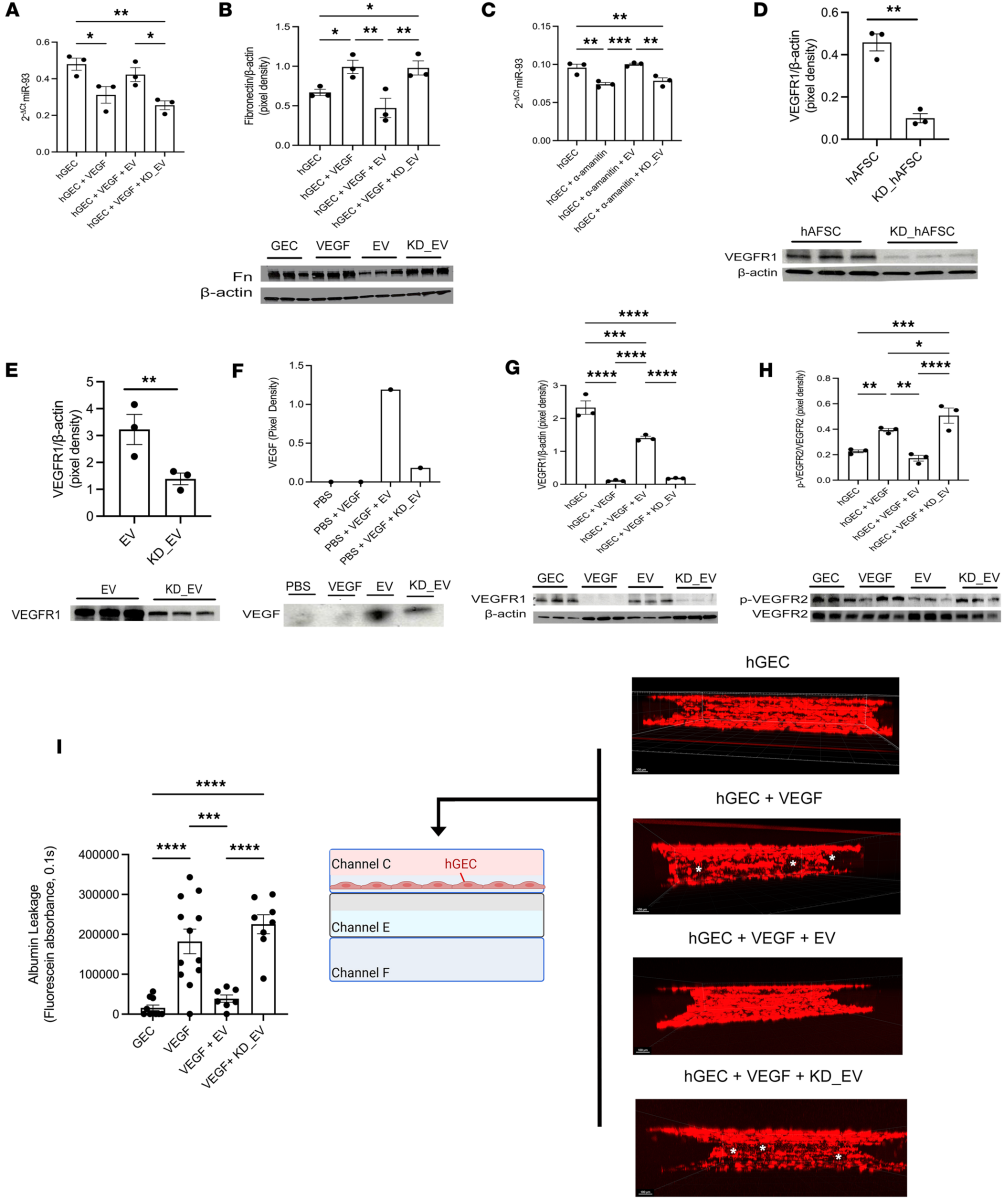
EVs regulate the miR-93/VEGFR1/VEGFR2 axis in GECs. (**A**) Relative miR-93 expression in GECs at baseline, VEGF-induced damage, VEGF-induced damage plus EVs, and VEGF-induced damage plus KD_EVs. Note: *P* = 0.0.55 between the VEGF-induced damage and the VEGF-induced damage plus EVs group. Small nuclear RNA U6 was used as a reference to calculate relative expression. (**B**) Densitometric analysis for fibronectin (261 kDa) by Western blot (WB) in GECs under indicated conditions. Quantification was normalized to β-actin (42 kDa). WB bands are shown below. (**C**) Relative miR-93 expression in GECs under indicated conditions. Small nuclear RNA U6 was used for normalization. (**D** and **E**) Densitometric analysis of VEGFR1 (75 kDa) in hAFSCs and KD hAFSCs (**D**) and in EVs and KD_EVs (**E**). For hAFSCs in **D**, quantification was normalized to β-actin (42 kDa). (**F**) Coimmunoprecipitation assay for VEGF (25 kDa) using an anti-VEGFR1 antibody on EVs and KD_EVs exposed to VEGF. Expression of VEGF by WB analysis as indicated. WB bands are shown below. (**G**) Densitometric analysis for VEGFR1 (75 kDa) in GECs under indicated conditions, normalized to β-actin (42 kDa). WB bands are shown below. (**H**) Densitometric analysis for p-VEGFR2/VEGFR2 (250 kDa and 192 kDa, respectively) ratio in GECs under indicated conditions. WB bands are shown below. (**I**) Left: Graph showing fluorescein absorbance (log scale) in the GEOAC in channel F (“filtrate”) after 60 minutes in GECs under indicated conditions (8–12 chips per group). Right: 3D confocal *Z*-stack images showing GEC monolayer formation, GECs with VEGF-induced damage, GECs with VEGF-induced damage plus EVs, and GECs with VEGF-induced damage with KD_EVs. GECs were labeled with CellTracker Deep Red. White asterisks represent disruption of the continuous monolayer in the damaged GEOAC and GEOAC treated with KD_EVs or EVs (scale bars: 100 μm). Data are reported as mean ± SEM. *P < 0.05; **P < 0.01; ***P < 0.001; ****P < 0.0001 by 1-way ANOVA with uncorrected Fisher’s LSD post hoc test (**A**–**C** and **G**–**I**) or unpaired, 2-tailed Student’s *t* test (**D**–**F**).

Since GECs are the primary source of miR-93 changes along disease progression, we evaluated the effects of EVs or KD_EVs in human GECs (hGECs). Following VEGF-induced damage, miR-93 expression was significantly reduced in hGECs and restored after exposure to EVs but not to KD_EVs (Figure 3A). Fibronectin expression, which has been shown to increase after VEGF-induced damage in hGECs (40) and is also a miR-93 target (28), was restored to normal levels when exposed to naive EVs (Figure 3B). Next, we evaluated whether miR-93 was transferred as part of the EV cargo to hGECs by reducing endogenous miR expression in hGECs using α-amanitin (an inhibitor of miR synthesis, ref. 41). We detected normal expression of miR-93 after treatment with EVs but not with KD_EVs, suggesting a possible direct transfer of miR-93 from EVs to hGECs (Figure 3C).

We previously demonstrated (11) that EVs can reduce the availability of VEGF because of the presence of VEGFR1 (42) on their surface that can trap VEGF. Therefore, we determined whether miR-93 regulates VEGF trapping by modulating the expression of VEGFR1. Silencing of miR-93 reduced expression of VEGFR1 in hAFSCs and EVs (Figure 3, D and E). We performed a coimmunoprecipitation assay using an anti-VEGFR1 antibody and then probed for VEGF, and showed a significantly higher efficiency of EVs in trapping VEGF versus KD_EVs (Figure 3F). We also demonstrated that miR-93 directly regulates VEGFRs in damaged hGECs since KD_EVs do not restore to normal the level of VEGFR1 expression and p-VEGFR2/VEGFR2 activity versus normal EVs (Figure 3, G and H). In addition, albumin leakage assay in the glomerulus/endothelium on-a-chip (GEOAC), generated using the same system as reported previously (43, 44), showed that EVs but not KD_EVs preserve the GEC barrier integrity in VEGF-induced damage (Figure 3I). Taken together, these data confirm that miR-93 modulates the expression of VEGFR1 and the activity of VEGFR2 in GECs and regulates GEC function.

We also investigated the effect of naive EVs versus KD_EVs in human podocytes (Supplemental Figure 6, A and B) and in mesangial cells in vitro (Supplemental Figure 6, C and D). In contrast with GECs and podocytes, damage to mesangial cells induced an upregulation of miR-93, which was restored with KD_EVs and not with normal EVs. This result is surprising but intriguing, as EVs appear to modulate damage in these cells through different mechanisms, possibly not involving a direct miR-93 action. Further investigations focused on understanding EV uptake by different glomerular cells and their cargo’s mechanism of action are needed, and are beyond the scope of this work.

### EVs regulate transcriptional changes in vivo.

To correlate changes in histological architecture with changes in gene expression during disease progression and to determine the disease modifying activity of EVs, we used Visium Spacial Transcriptomics (ST) (Supplemental Figure 7, Supplemental Methods, and GEO GSE245039) in WT (4m), 2mAS mice, 5mAS mice, and AS mice injected with hAFSC-EVs at 2.5 months (before high level of proteinuria) and analyzed at 5 months. Histological analysis showed scarring in 5mAS versus 2mAS and WT mice, which was attenuated in injected mice (Supplemental Figure 8, A–D). Each sample was analyzed individually, and spatial maps with cluster annotations and Gene Ontology (GO) analysis are reported in Supplemental Figure 8, E–P, and Supplemental Datasets 4–7.

To note, because of the size and spacing of the ST spot (55 μm spots spaced 100 μm apart), multiple cell types are included within each spot; therefore, each cluster is composed of multiple renal structures. Nevertheless, the cluster identity was confirmed by histological observation and based on the cell-specific genes found elevated within each cluster.

**Figure 4:**
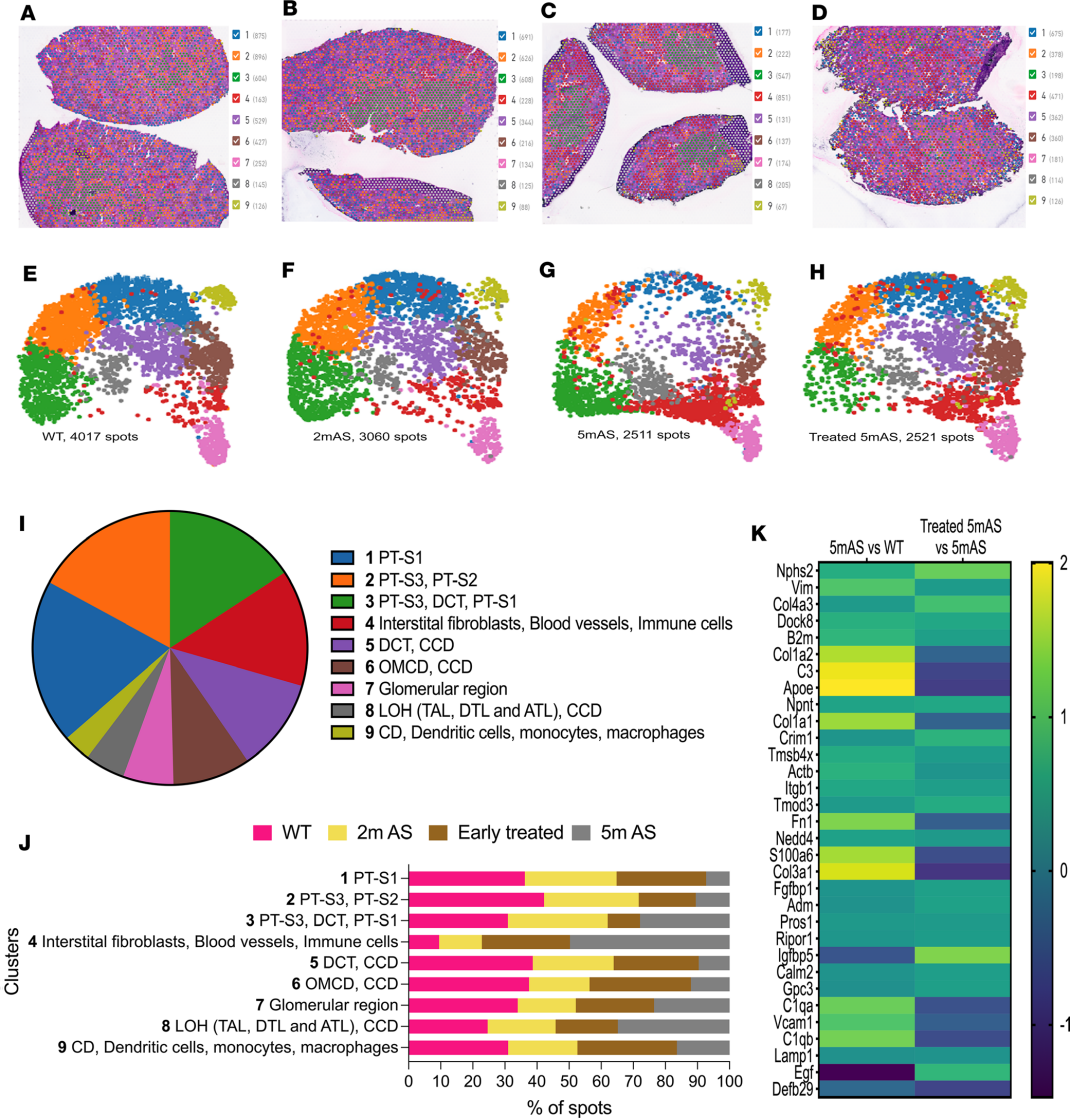
Spatial maps of kidney of AS mice injected with EVs. (**A**–**D**) ST integrated analysis of WT mice at 4 months old (4m), 2mAS, 5mAS, and hAFSC-EV injected at 2.5m and sacrificed at 5mAS (treated 5mAS), identified 9 clusters by unsupervised clustering as shown in the spatial map for each sample. (**E**–**H**) UMAP of spots from the integration of samples in **A**–**D**, showing the cluster identification per sample (**I**) Pie chart displaying the distribution of dots across clusters of the integrated samples. Total dots = 12,453. (**J**) Graph displaying the distribution of samples across clusters. Number of spots (*x* axis) of each sample (magenta, WT; yellow, 2mAS; gray, 5mAS; brown, treated at 2m and sacrificed at 5mAS) per cluster identified (*y* axis). (**K**) Heatmap showing fold change (FC) in gene expression of miR-93 target genes in glomeruli: 5mAS vs. WT and treated 5mAS vs. 5mAS showing a significant shift in gene expression following EV injection. Heatmap shows log_2_FC calculated relative to the first condition listed in each comparison: 5mAS vs. WT and treated 5mAS vs. 5mAS. Color intensity reflects the magnitude of differential expression relative to the indicated reference condition. log_2_FC > 0 or log_2_FC < 0; *P* < 0.05. Yellow, upregulated;blue, downregulated. PT-S1, -S2, -S3, proximal tubule segments 1, 2, and 3; DCT, distal convoluted tubule; CCD, cortical collecting duct; OMCD, outer medullary collecting duct; LOH, loop of Henle; TAL, thick ascending limb; DTL, descending thin limb; ATL, ascending thin limb; CD, collecting duct. Significance was assessed using differential expression determined by DESeq2 (adj. *P* < 0.05).

Unsupervised clustering of integrated samples identified 9 clusters (Figure 4, A–H). Cluster annotation and sample contribution per cluster are shown in Figure 4, I and J, and Supplemental Dataset 8, and the most highly expressed genes in each cluster are reported in Supplemental Figure 9). All samples contributed to all the clusters, but there was a clear shift in cluster distribution in 5mAS, with clusters 1, 2, 5, and 6 being overly underrepresented and cluster 4 (immunological and fibrotic interstitium) specifically being overrepresented, whereas the treated and the 2mAS showed similar cluster distribution to the WT (Figure 4J).

**Figure 5:**
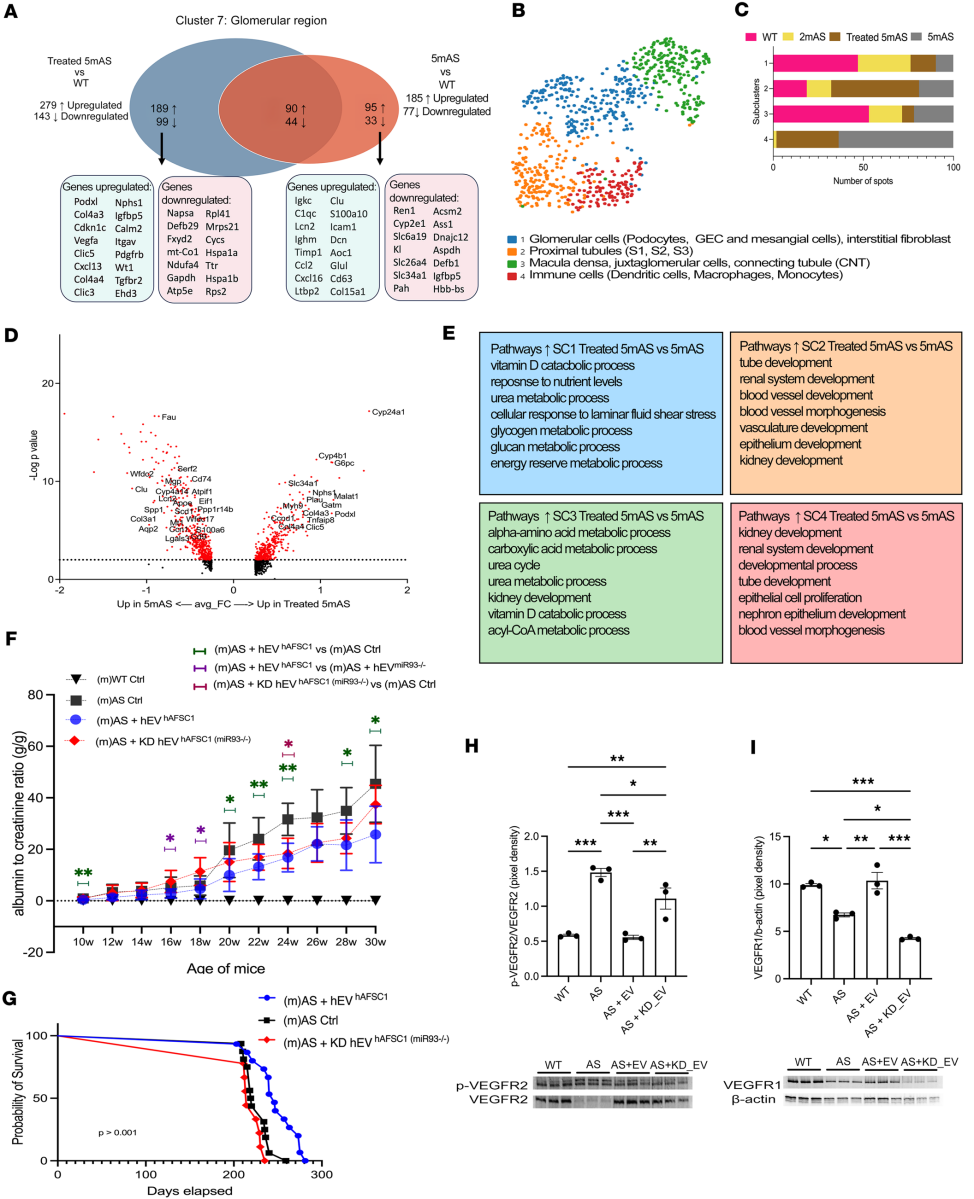
Spatial maps of glomeruli of AS mice injected with EVs. (**A**) Venn diagram displaying significantly upregulated genes in glomerular spots of treated 5mAS vs. WT at 4m (left) and 5mAS vs. WT 4m (right). Treated 5m mice received hAFSC-EVs at 2.5m and sacrificed at 5m. Significantly regulated genes (|log_2_FC| > 0.25; adj. *P* < 0.05). (**B**) Glomerular cluster (cluster 7 from the integrated samples in Figure 4) was filtered, reclustered, and subdivided into 4 different subclusters by unsupervised clustering. Cluster annotations are shown on UMAP. (**C**) Distribution of samples across glomerular subclusters. Percentage of glomerular spots (*x* axis) of each sample (magenta, WT 4m; yellow, 2mAS; gray, 5mAS; brown, treated 5mAS) per subcluster identified (*y* axis). (**D**) Volcano plot of DEGs in treated 5mAS vs. 5mAS within subcluster 4 of immune cells (|log_2_FC| > 0.25; adj. *P* < 0.05). (**E**) Enriched Gene Ontology (GO) biological processes obtained from significantly upregulated genes in glomerular spots of treated vs. untreated 5mAS mice, for subclusters sc1 (blue), sc2 (orange), sc3 (green), and sc4 (red). (**F**) Proteinuria assessed by albumin-to-creatinine ratio, in AS mice following injection of either EVs (blue, *n* = 16) or KD_EVs (red, *n* = 11) at 2.5m. Untreated AS mice (black, *n* = 15), and WT mice (black, *n* = 5) served as controls. (**G**) Survival curve of AS mice, untreated (black, *n* = 15) or treated with EVs (blue, *n* = 16) or KD-EVs (red, *n* = 11). (**H**) Densitometric analysis for p-VEGFR2/VEGFR2 ratio (250 kDa and 192 kDa, respectively) in glomeruli of indicated groups. (**I**) Densitometric analysis for VEGFR1 (150 kDa) in glomeruli of of indicated groups; normalized to β-actin (42 kDa). Western blot bands are shown. Data are reported as mean ± SEM. *P < 0.05; **P < 0.01; ***P < 0.001 by 1-way ANOVA with uncorrected Fisher’s LSD test for proteinuria (**F**, **H**, and **I**) or log-rank (Mantel-Cox) test for survival curves (**G**).

Since AS is a disease of the glomerulus, we looked for the transcriptional changes of downstream targets of miR-93 in cluster 7 (the glomerular cluster). We observed upregulation of *Fn1* and downregulation *of Nphs2*, *Itgb1*, and *Vim*, which were reversed by human EV administration (Figure 4K). The complete list of the miR-93 targets between the samples is reported in Supplemental Dataset 9. GO analysis of upregulated genes identified regenerative pathways in EV-treated mice versus nontreated mice (Supplemental Figure 10A). Injection of EVs downregulated apoptosis and stimulated changes in metabolic pathways and renal regeneration (Supplemental Figure 10B). A complete list of the pathway analysis is reported in Supplemental Dataset 10. The distribution of differentially expressed genes (DEGs) between EV-treated and non–EV-treated glomeruli compared with WT identified 189 genes that were exclusively upregulated in treated mice (Figure 5A), including *Podxl*, *Cdkn1c*, *Nphs1*, and *Wt1*, and upregulation of pathways related to VEGF binding, ECM binding, and glomerular development, while the non–EV-treated glomeruli showed upregulation of pathways like misfolded protein, cell death, L13a-mediated translational silencing of ceruloplasmin expression, SRP-dependent cotranslational protein targeting to membrane, pathways also altered in human AS glomeruli, as we published previously (45). These pathways were downregulated in treated mice, showing the potential therapeutic effect of EVs (Supplemental Figure 11A and Supplemental Dataset 11). The distribution of DEGs between EV-treated, non–EV-treated, 2mAS glomeruli versus WT (Supplemental Figure 11B and Supplemental Dataset 12) showed 181 genes exclusively upregulated in treated mice, with enrichment in pathways related to development. A complete list of the pathway analysis is reported in Supplemental Dataset 13.

To deepen our analysis, we reclustered the glomerular spots and identified 4 subclusters (Figure 5B, Supplemental Figure 12, and Supplemental Dataset 14). The enrichment for tubular genes was not surprising based on the resolution of ST, as specified above. Subclusters 1 and 3 were mainly represented by the WT and 2mAS, subcluster 2 by the treated mice, and subcluster 4 almost exclusively by the nontreated and treated AS mice (Figure 5C).

To understand the effects of the EVs in glomerular subclusters, we analyzed the DEGs in treated versus nontreated 5mAS mice (Figure 5D and Supplemental Dataset 15) and identified enrichment of pathways involved in renal development and in lipid metabolism, which were underrepresented in AS (Figure 5E). A complete list of the pathways analysis is reported in Supplemental Dataset 16.

We then injected AS mice before the onset of heavy proteinuria with either EVs or KD_EVs; normal EVs showed significant improvement in proteinuria versus untreated AS mice or mice treated with KD_EVs. Even if mice injected with KD_EVs showed some improvement in proteinuria, they did not increase survival versus mice treated with normal EVs (Figure 5, F and G). We also showed that in vivo, naive EVs can restore to normal the expression of VEGFR1 and the activity of VEGFR2 versus KD_EVs (Figure 5, H and I).

These data confirm that EVs present with disease-modifying activity and that possibly miR-93 is involved in regulating this renoprotection.

### miR-93 targets are also altered in GECs of patients with AS.

**Figure 6:**
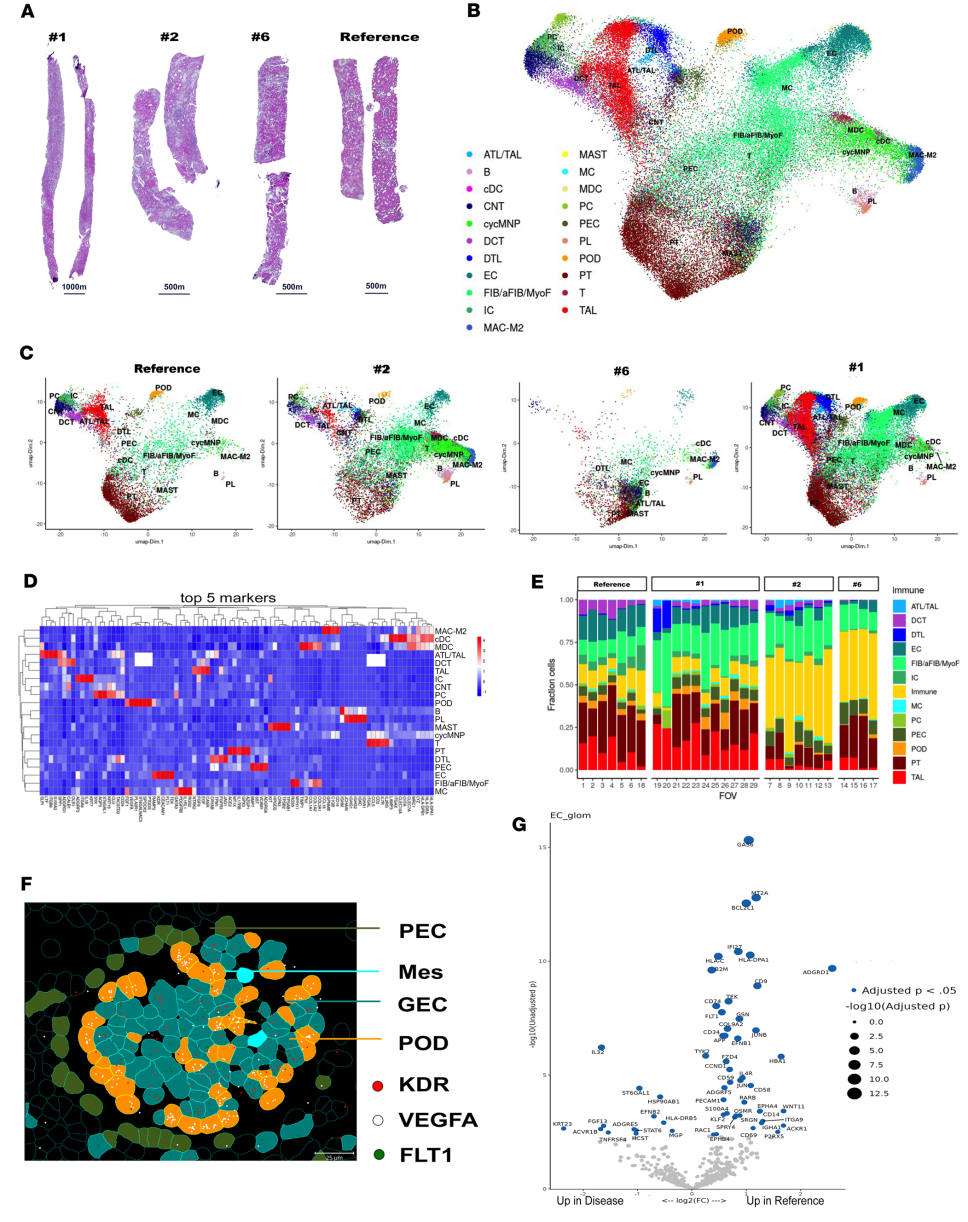
Spatial maps of AS biopsies. (**A**) Histology micrographs from serially sectioned kidney biopsies from a 3-year-old patient with AS (no. 1), a 36-year-old patient with AS (no. 2), a 42-year-old patient with AS (no. 6), and a 19-year-old patient used as reference were stained for H&E followed by FOV section and CosMx SMI analysis. (**B** and **C**) UMAP visualization of cell classification based on the integration of the 4 biopsies (**B**) and for individual biopsy (**C**). A total of 29 FOVs were included in the final analysis following QC, distributed among 21 cell types as indicated by color codes. (**D**) Heatmap showing expression of top 5 markers identifying the renal cell populations classified in **B**. (**E**) Cell deconvolution analysis showing the abundance of different cell types in the 29 FOVs from biopsies shown in **A**. Analysis was based on publicly available RNA-seq experiments compiled into profile matrices by aggregating gene-wise counts of all annotated cell types. (**F**) Representative glomerulus from nondiseased patient showing cell segmentation and identification of podocytes (orange), GECs (blue), mesangial cells (light blue), and parietal epithelial cells (green). mRNA probe for VEGFA (white dots), KDR (red dots), and FLT1 (green dots) are spatially localized in the podocytes (VEGFA) and in GECs (KDR and FLT1). Cells that failed to pass the QC are represented in black. Scale bar: 25 μm. Images were visualized and captured using Napari 4.0.17 software. (**G**) Volcano plot showing the DEGs in GECs from all biopsies combined vs. reference (|log_2_FC| > 0.2; adj. *P* < 0.05). Statistical analysis was done to determine differential expression by DESeq2 (adj. *P* < 0.05). ATL, ascending thin limb; TAL, thick ascending limb; B, B lymphocytes; cDC, classical dendritic cells; CNT, connecting tubules; cycMNP, cycling mononuclear phagocytes; DCT, distal convoluted tubules; DTL, descending thin limb; EC, endothelial cells; FIB/aFIB/MyoF, fibroblasts/adaptive fibroblasts/myofibroblasts; IC, intercalated cells; MAC-M2, type 2 macrophages; MAST, mast cells; MC, mesangial cells; MDC, monocyte-derived cells; PC, principal cells; PEC, parietal epithelial cells; PL, plasma cell; POD, podocytes; PT, proximal tubules; T, T lymphocytes.

To translate our data from mouse to human, we applied the CosMx Spatial Molecular Imaging (SMI) platform in biopsies of patients with AS, which allows for the spatial mapping of gene expression at single-cell resolution (46). We studied 3 different patients with AS with known *COL4A5* mutations, and we used a biopsy from a transplanted kidney as a reference (Figure 6A and Supplemental Figure 13A). We manually selected 29 fields of view (FOVs), capturing all the glomeruli available (Supplemental Figure 13B). We performed stringent quality control (QC) by eliminating cells with fewer than 20 transcripts (cells that did not pass QC are represented in black in the segmentation analysis). Using the NanoString pipeline and data available (47), we identified 21 cell types (Figure 6, B–D); the mean of the confidence score is shown in Supplemental Figure 13C. Cell deconvolution analysis displayed an abundance of the major glomerular, tubular, and interstitial cells (Figure 6E); a cell type representation for each FOV is shown in Supplemental Figure 14A. Abundances of immune cell types were detected between diseased and nondiseased FOVs, especially in 2 patients with AS, nos. 2 and 6, who both presented arteriolosclerosis, and no. 6 also low-grade IgA nephropathy (Figure 6E; for clarity, the immune cells here were grouped together). We focused on the glomerulus and, using Napari (48), a multidimensional image viewer for Python, we identified the spatial localization of the glomerular cells (Figure 6F, Supplemental Figure 14B, and Supplemental Figure 15). We confirmed that this technology can spatially recognize gene expression at the cellular level by showing that VEGF is expressed in podocytes, and its receptors KDR and FLT1 are expressed in GECs (Figure 6F).

Based on our stringent QC, which reduced the number of cells available for glomerular analysis, we could not quantify gene expression differences for all comparisons for all glomerular cell types/samples. We could confidently only perform analysis on GECs after combining all 3 AS biopsies versus the reference. Despite these limitations, our data confirmed that AS-GECs presented with loss of endothelial markers (*PECAM*, *CD34*, and *TEK)* and showed upregulation of *IL32*, *FGF1*3, and *MPG* (Figure 6G). Interestingly, AS-GECs seem to present strong downregulation of the *FLT1* gene, which encodes both VEGFR1 and its soluble form sFLT1. Loss of sFLT1 can induce an unbalanced accumulation of VEGF produced by the podocytes, thus causing glomerular damage. These findings confirm target engagement in human AS glomeruli; for example, miR-93 downregulation and *FLT1* gene loss were consistently detected in hGECs. Translationally, this suggests that EVs enriched in miR-93 could restore VEGF signaling homeostasis in the human setting. Identifying primary assays critical for characterizing the final EV product in a therapeutic context is paramount for regulatory approval for clinical translation.

Based on our preclinical in vitro and in vivo work and EV characterization derived from an established clonal population of AFSCs, together with the evidence that the major EV targeting signaling (VEGF/miR-93) is present in human AS glomeruli, we can envision a stable set of parameters for our EVs that can guarantee EV lot production with the same characteristics. We can design identity and purity parameters identified by the expression of specific markers, as in Figure 1, but most importantly, we can define a mechanism of action by a potent assay based on the presence of miR-93 and its capability of regulating VEGF signaling. Finally, we can design a disease-modifying activity that can be assessed by lowering proteinuria and by stabilizing the slope of the estimated glomerular filtration rate in patients with AS.

## Discussion

EVs are an important cell-to-cell communication system, able to deliver regenerative factors from stem cells to target cells. The mechanisms involved in their therapeutic effects are numerous; they may interact with targeted cells through surface membrane receptors, deliver growth factors present in their corona, or be internalized and act as trophic factors or deliver a specific cargo (19, 49–52). We previously identified that AFSC-EVs could act as decoy factors, trapping VEGF through surface VEGFRs, thus modulating podocyte-GEC crosstalk by attenuating VEGF-induced glomerular injury (11).

In continuing our effort to understand the VEGF-modulating effects of AFSC-EVs in AS, here we characterized the EV cargo, with a specific focus on the miRs, which represent a major component of EV cargo and are modulators of gene expression (19, 53). We previously showed (11) that hAFSC-EVs contain miRs with angio-modulatory properties regulating key components of VEGF signaling, including VEGF (miR-93 and -16.1), VEGF receptors (miR16.1), and different upstream and downstream targets of this signaling (miR-23a, -27a, -221, 322, and -145). Interestingly, miR-93 (miR-93-5p), a key modulator of VEGF signaling (28), compared with all the other angio-modulatory miRs, is highly expressed in our EVs.

Therefore, based on the evidence that miR-93 highly regulates VEGF signaling and that it is highly expressed in EVs, here we investigated whether miR-93 EV–specific cargo transfer would affect EV functional properties in rescuing glomerular injury, since our data showed that miR-93 expression is highly reduced in AS glomeruli and in biopsies of patients with AS.

We first determined that silencing of miR-93 in EVs induced a striking shift in the levels of various miRs (and their targets) and proteins that are critical for glomerular cell function, thus impairing EV renoprotective activity. We observed an upregulation of miR-432-5p, which targets *COL4A5* and fibronectin, important for ECM remodeling and AS disease progression (54, 55). To note, even if *COL4A5* genes emerged among the predicted miR targets that we identified, in our study, a modulation of this gene might be part of the ECM remodeling process, rather than a direct therapeutic correction. Similarly, miR-10b-5p, which targets *ITGb8* (involved in cell-matrix adhesion; ref. 56), was also upregulated. miR-135b-5p, targeting *COL4A3*, and miR-137, targeting *PDGFRa* (important for mesangial cell function; ref. 57), were elevated. Concomitantly, we noted a downregulation of key proteins involved in maintaining glomerular architecture and cellular integrity. Loss of miR-93 alters molecular signaling affecting actin polymerization, cadherin binding, collagen trimer formation, and cell-matrix adhesion–related functions, thus suggesting that miR-93 plays a pivotal role in shaping the EV cargo composition and impacts pathways relevant to AS pathogenesis. The importance of miR-93 in regulating AS disease progression was also validated in ST studies. We identified changes in expression of miR-93 targets (like *Tgfbr2*, *Mmp2*, *Col4a3*) specifically in the glomerulus that were restored to normal after EV administration. Most importantly, EVs lacking miR-93 were significantly less efficient in rescuing kidney function in vivo, demonstrating that miR-93 is an important contributor to EV-mediated protection of glomerular homeostasis, although different cargo components (19, 20) and mechanisms likely cooperate in this effect.

The renoprotective role of miR-93 has been reported in other CKDs (58–62) and in an animal model of acute myocardial infarction (63), and it is known that miR-93 directly modulates VEGF expression in podocytes (28) in hyperglycemic conditions.

Interestingly, our data suggest that in AS, miR-93 downregulation is specifically driven by GECs, suggesting a cell-specific mechanism for the dysregulation of miR-93, not involving podocytes or mesangial cells. Mechanistically, we showed that EVs regulate both the expression of VEGFR1 and the activity of VEGFR2 directly in GECs, demonstrating that EVs can modulate VEGF signaling not only by reducing the bioavailability of VEGF produced by podocytes within the glomerular space (11) but also by influencing the receptor activity. Our GEOAC experiments also showed that EVs modulate GEC function by regulating their barrier properties in VEGF-induced damage. Most importantly, we showed that this angio-modulatory effect is regulated by miR-93.

Our studies pointed to miR-93/VEGFR axis regulation in GECs as one possible mechanism of action of EVs and even if it is recognized that EVs induce different effects, our results highlight how disease modulators, like miR-93, might be specific to one cell type in the glomerulus (in GECs at least in AS), since miR-93 expression is not altered in podocytes and mesangial cells during disease progression. These data have important implications for future therapeutics; even if miRs or anti-miRs have been tested in clinical applications (64, 65), including the use of anti–miR-21 (66) in AS, the systemic delivery of naked miRs will not likely be effective in regulating changes specific to one cell type in the glomerulus (or in other cell types). EVs are stable in circulation and protect their cargo, including miRs, from degradation and might reach targeted cells in an effective manner (19, 21, 23, 52). Therefore, we believe that the next generation of EV therapeutics should include disease-specific, cell-targeted EVs, which will allow the generation of effective therapeutic responses where needed.

From a translational perspective, these data highlight how an EV-based therapy might complement current standards of care. By demonstrating that EVs act specifically through miR-93 cargo to restore endothelial VEGF signaling, our findings provide a clear mechanism of action and a measurable marker of target engagement that can guide trial design, while acknowledging that further studies are required to fully establish efficacy, optimize dosing, and clarify the contribution of additional EV cargo.

Our study presents some limitations. ST resolution was limited to multiple cells per spot due to the larger diameter of the spots. Thus, the cell type classification did not achieve single-cell resolution. To overcome this limitation, we subclustered the spots identified as glomerular, and we could evaluate a shift in glomerular cell populations in each sample. We acknowledge the limited number of samples used in the ST studies, but we believe multiple in vivo and in vitro studies support the finding of the ST. Given that KD_EVs failed to reverse damage in our in vitro and in vivo experiments, we did not proceed with a full spatial analysis for the KD_EV condition, but prioritized profiling of untreated AS mice and those treated with hAFSC-EVs at different time points to investigate the baseline disease trajectory and therapeutic potential of EV-delivered miR-93. We recognized the limitations of CosMx; this platform allowed only for 1000 genes (at the time when the analysis was performed), and our QC excluded multiple cells, reducing our ability to analyze DEGs in all cell types. Nevertheless, we used 3 different biopsies with *COL4A5* mutations that allowed us to determine changes in human AS GECs. The impact of miR-93 effects within other renal cells remains to be further elucidated, since here we focused on GECs. While the therapeutic effect of the EVs is noticeable in male mice, particularly in our AS colony, it is important to test it also in other AS strains along with female mice.

In summary, we demonstrated that miR-93 exerts key effects on the progression of AS, with GECs emerging as the primary target of miR-93–mediated actions. While our understanding of the mechanism of action of EVs remains to be completed, we suggest that miR-93 plays a predominant role in mediating the EVs’ effects. Based on these collective findings, we believe that EV-based therapies might hold promise as a possible complementary approach to standard-of-care treatment for patients with CKD.

## Methods

### Sex as a biological variable.

All animal experiments were conducted using male mice since the *COL4A5* mutation is an X-linked mutation. In addition, all our previous animal studies were performed in the same male animal model, allowing for direct comparison with existing experiments and thus minimizing variability between datasets. Future animal studies will be necessary to determine whether the results presented here are also evident in heterozygous females and in the *Col4a3* and *Col4a4* mutations. For human biopsies, we did not consider sex differences and analyzed the biopsies that were available with known *COL4A5* mutations.

### Animal studies and mouse cell isolation.

AS mice (*Col4a5^–/–^*; B6.*Cg-Col4a5^tm1Yseg^*/J) and WT mice (C57BL/6J) were purchased from The Jackson Laboratory and bred as published previously (11). Glomeruli and glomerular cells were isolated as published previously (11), and in vivo experiments are described in Supplemental Methods. The gating strategy for cell isolation is shown in Supplemental Figure 1.

### hAFSC culture, EV isolation and characterization, in vitro experiments.

hAFSCs were isolated and cultured as previously described (11, 29–31) and EVs isolated as previously described (11) to avoid bovine EV contamination. EV size, number, TEM, super-resolution microscopy (ONI), EV miR sequencing, and EV proteomics were performed using standard protocols and details are described in the Supplemental Methods. Isolation and culture of human glomerular cells was performed as previously described (43, 44), and protocols for Western blotting, in situ hybridization, and coimmunoprecipitation are reported in Supplemental Methods.

### miR-93 quantification, KD_EV generation, in vitro damage, α-amanitin experiments, and GEOAC experiments.

To detect miR-93 expression in mouse samples, the TaqMan miRNA Reverse Transcription kit and the cDNA preamplified with TaqMan PreAmp Master Mix (Thermo Fisher Scientific, 4384267) were used. qPCR was performed using the TaqMan microRNA Assay (Thermo Fisher Scientific). For human samples, total RNA was extracted from paraffin tissue sections using an FFPE RNA Purification Kit (Norgen Biotek Corp., 25400). Small nuclear RNA U6 was used to normalize qPCR results across samples.

KD_EVs were generated by incubating 1 × 10^6^ hAFSCs with miR-93-5p antagomiR (Applied Biological Materials, MNH03941; 1 nmol/10^6^ cells, transient KD) in lipofectamine (Invitrogen, 13778150; 100 μL/10^6^ cells) for 6 hours and EVs isolated as in Sedrakyan et al. (11). The efficiency of miR-93-5p KD in EV cargo was validated by qPCR. To induce cell type–specific damage, 100,000 cells/well were used. GECs were exposed to VEGF (Thermo Fisher Scientific, Phc9394; 400 ng/mL, 3 days), mesangial cells to TGF-β (R&D Systems, 240-B-002; 1 mg/mL, 3 days), and podocytes to puromycin (Sigma-Aldrich, SBR00017; 50 μg/mL, 5 days). To reduce miR expression, hGECs (100,000 cells/well) were incubated with α-amanitin (Sigma-Aldrich, A2263-1MG; 50 μg/mL) with hAFSC-EVs or KD_EVs (1 cell/10^4^ EVs ratio) for 3 hours; 5 μg of total RNA was used to evaluate miRNA expression by RT-PCR.

GEOACs were generated as detailed in the Supplemental Methods. hGECs (30,000 cells) were seeded into the top channel (channel C) inlet at a density of 1.5 × 10^7^ cells/mL. Five days after hGEC seeding, cells were treated with VEGF (400 ng/mL, 6 hours; Thermo Fisher Scientific, Phc9394) to induce injury and with hAFSC-EVs or KD_EVs (1 cell/10^4^ EVs ratio). hGEC barrier function was assessed by albumin absorbance in filtrate collected from channel F as described previously (43, 44).

### Transcriptomics studies.

Bulk RNA-seq was performed to determine changes in gene expression and miR-93 targets in AS versus WT glomeruli, while Visium ST (10x Genomics) was used to investigate the renoprotective role of EVs in AS and to determine changes in gene expression along disease progression. SMI (CosMx, NanoString) was performed to evaluate changes in gene expression in a human setting. Detailed description of the procedures and data analysis are described in the Supplemental Methods.

### Statistics.

Data are expressed as mean ± SEM. For comparisons between 2 groups, an unpaired 2-tailed Student’s *t* test was used. For multiple group comparisons, 1-way ANOVA with uncorrected Fisher’s LSD post hoc test was applied unless otherwise specified. Survival curves were analyzed using the log-rank (Mantel-Cox) test. For transcriptomics and proteomics datasets, DEG analysis was performed using DESeq2 (see Supplemental Methods), with an adjusted *P* value of less than 0.05 considered significant. A *P* value of less than 0.05 was considered statistically significant throughout. Statistical analysis for the transcriptomics and proteomics data is described in the Supplemental Methods.

### Study approval.

AFSCs were obtained as described previously (11, 29–31), and samples were approved by the Children’s Hospital of Los Angeles (CHLA) IRB; exemption was obtained since no consent was required, as samples were deidentified. Samples of amniotic fluid presented with a normal karyotype and confirmed negative for infectious diseases. Discarded kidneys, CHLA IRB approved, were harvested from patients with a non-nephrological cause of death, allowing functional cells for our in vitro studies. For studies of human AS biopsies and healthy tissue biopsies (for Figure 1D, reference no. 1 and AS no. 2; Figure 1E, reference no. 3; and for Figure 6 biopsies are described in B) were obtained from the pathology biorepository at the Department of Pathology and Lab Medicine, David Geffen School of Medicine at UCLA. The IRBs of CHLA and UCLA approved the protocol for the use of archived human samples. All experiments were performed in accordance with ethical guidelines and regulations of the Declaration of Helsinki. Animal studies were performed according to the NIH *Guide for the Care and Use of Laboratory Animals* (National Academies Press, 2011) and CHLA IACUC approval.

### Data availability.

The data supporting the results of this study, including bulk-RNA seq, spatial transcriptomics, and EV analysis cargo, are available from the corresponding author. Raw data and statistics for these experiments are provided in Supplemental Datasets 1–17, Supplemental Methods, Supporting Data Values file, and GEO GSE245039.

## Authors contributions

CD performed experiments for all the experiments, analyzed data, and wrote the manuscript. VV analyzed data and wrote the manuscript. XH conducted experiments for glomeruli collection. PN performed EV isolation and characterization. GC performed and analyzed proteomics data. MK conducted GEOAC experiments. CG conducted EV TEM and EV ONI experiments. PC analyzed data and wrote the manuscript. PA conducted cell culture, EV isolation, and data analysis. VA performed in situ hybridization and data analysis. GO provided hGECs and analyzed data. XYS performed ST and analyzed ST data. JEZ provided biopsy and clinical data of patients with AS and references, and revised the manuscript. REDF analyzed data and revised the manuscript. SDS performed GEOAC experiments and performed miR EV sequencing. SS performed bulk RNA-seq and CosMx analyses and revised the manuscript. BB and LP conceived the project, analyzed data, and wrote the manuscript.

## Funding support

This work is the result of NIH funding, in whole or in part, and is subject to the NIH Public Access Policy. Through acceptance of this federal funding, the NIH has been given a right to make the work publicly available in PubMed Central.

- NIH grant 1R01DK121037-01A1 (to LP).
- The GOFARR Fund.
- The Extracellular Vesicles Core Pilot at CHLA.
- FACS Core Pilot at CHLA.

## Supplementary Material

Supplemental data

Supplemental data set 1

Supplemental data set 10

Supplemental data set 11

Supplemental data set 12

Supplemental data set 13

Supplemental data set 14

Supplemental data set 15

Supplemental data set 16

Supplemental data set 17

Supplemental data set 2

Supplemental data set 3

Supplemental data set 4

Supplemental data set 5

Supplemental data set 6

Supplemental data set 7

Supplemental data set 8

Supplemental data set 9

Unedited blot and gel images

Supporting data values

## Version 1 03/23/2026

Electronic publication
