# Supplementary material for: Extracellular vesicle miR-93-5p cargo regulates glomerular endothelial cell damage in Alport syndrome
Source: JCI Insight. 2026 Mar 23;11(6):e197643. doi: 10.1172/jci.insight.197643 (PMC13043101; doi:10.1172/jci.insight.197643)
Supplement: Supplemental data [file jciinsight-11-197643-s146.pdf]

Supplementary Materials

Extracellular vesicle-miR-93-5p cargo regulates glomerular endothelial cell damage in Alport syndrome.

Charmi Dedhia, Valentina Villani, Xiaogang Hou, Paolo Neviani, Geremy Clair, Mohammed Kasravi, Cristina Grange, Paolo Cravedi, Paola Aguiari, Velia Alcala, Giuseppe Orlando, Xue-Ying Song, Johnathan E Zuckerman, Roger E De Filippo, Stefano Da Sacco, Sargis Sedrakyan, Benedetta Bussolati, Laura Perin

Supplementary Figures 1-15

Supplementary Methods

Supplementary References for Methods

Supplementary Figures 1-15

Supplementary Figure 1. Gate Strategy for mouse glomerular cell isolation.

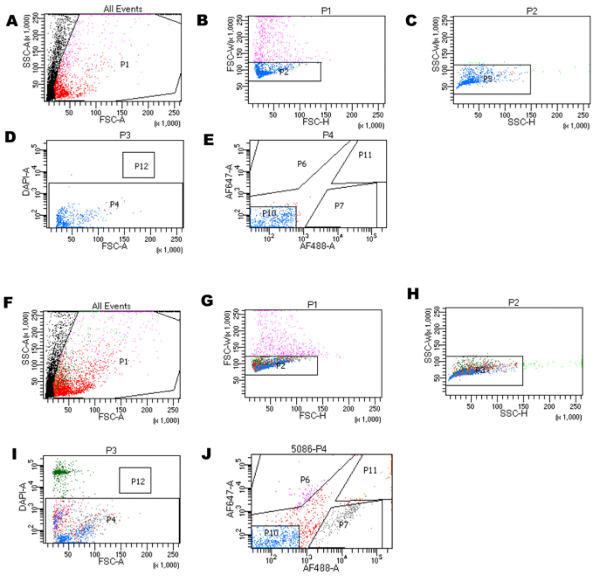

A. Cells were first gated based on forward scatter (FSC-A) and side scatter (SSC-A) areas to exclude dead cells and potential cellular debris from the analysis (P1). **B-C**. Further gating was performed to remove duplets based on FSC-H/FSC-W (P2) and SSC-H/SSC- W (P3). **D.** Gating was performed on DAPI. **E**. Podocytes were selected based on nephrin expression (AF-647, P6), GEC were selected based on VE-Cadherin expression (AF-488, P7), while cells negative for both nephrin and VE-cadherin were identified as mesangial cells (P10). **F-J.** Gating was performed following the same criteria but independently for each sample to reflect differences between the analyzed population (f-j).

Supplementary Figure 2. miR-93-3p expression in mouse glomerular cells and in human AS cortex and in situ hybridization controls.

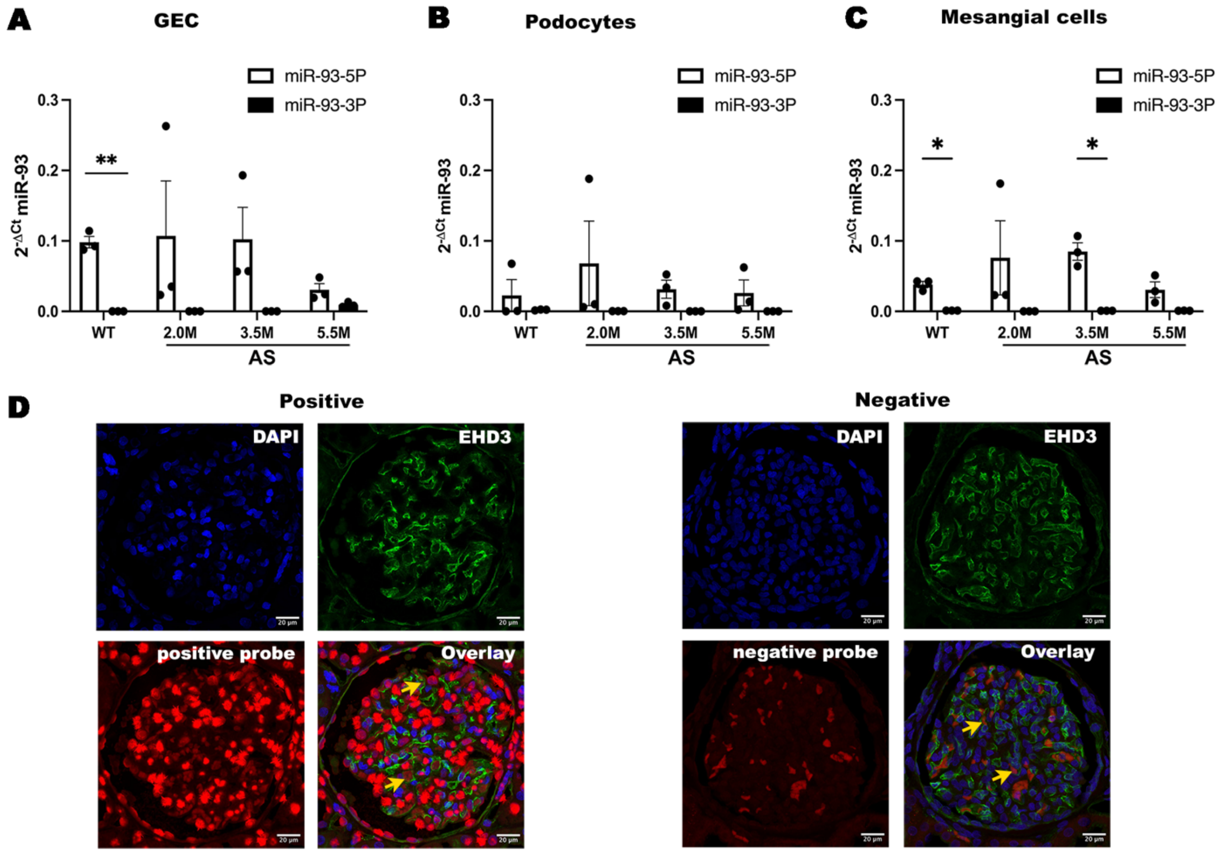

A-C. Graph showing miR-93-5p and miR-93-3p expression in GEC **(**A**)**, podocytes **(**B**),** and mesangial cells **(**C**)** isolated from WT (4m old) and AS mice at 2m, 3.5m, and 5.5m old (n=3). For miR-93 expression, Small nuclear RNA U6 was used as a reference to calculate relative expression, and all experiment was run in triplicate. All values are reported as mean ± SEM. Significant values are indicated as: * (p<0.05), ** (p<0.0 1), **** (p<0.0001). **D**. Representative images of human glomeruli, healthy tissue of a partial nephrectomy showing positive (left) and negative (right) for miR-93 expression by in situ hybridization. Upper left corner: DAPI in blue; Upper right corner: EHD3 in green, identifying GEC; lower left corner: miR-93 probe in red; Lower right corner: merged panels. White arrows: GEC positive for EHD3; yellow arrows: red blood cells (autofluorescence in red). Scale bar = 20μm. All values are reported (A-C) as mean ± SEM. Statistical significance was assessed using One-way ANOVA with post hoc uncorrected Fisher's LSD. Significant values are indicated as: *p<0.05, **p<0.01, ***p<0.001, ****p<0.0001.

Supplementary Figure 3. Bulk-RNA seq on male AS glomeruli.

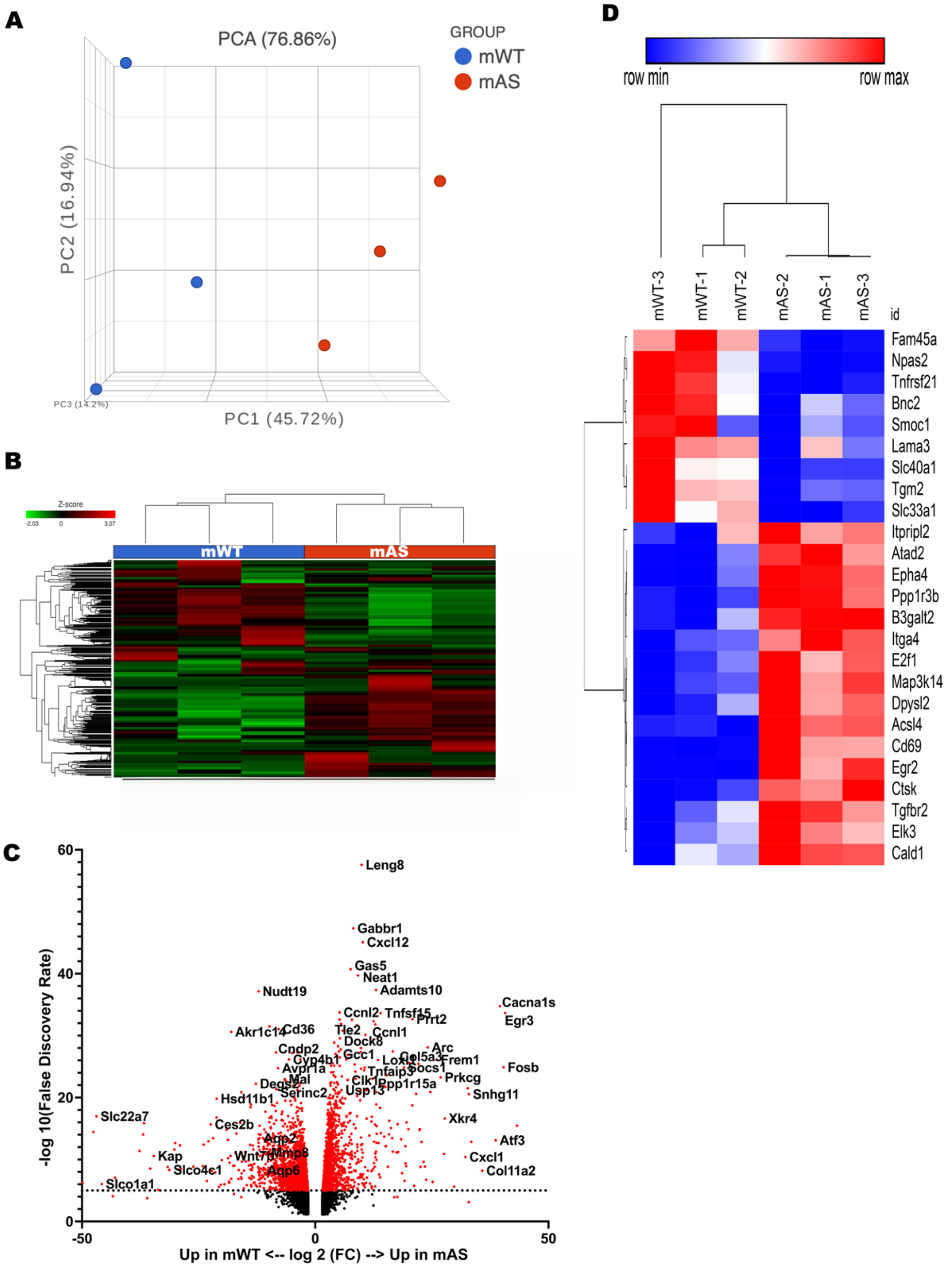

A. Principal component analysis (PCA) from glomeruli of WT mice (blue, n=3, male, 4m old) and AS mice (red, n=3, male, 4m old) showing the distribution of samples along PC1 (45% of the variance) and PC2 (16% of the variance). **B.** Hierarchical clustering heatmap representing DE genes in WT (blue group, left) and AS (red group, right), showing a shift with AS disease. **C.** Volcano plot showing the DE genes in AS *vs* WT (FC>1.5 or FC<-1.5, p-value<0.05). **D.** Hierarchical clustering heatmap showing FC in gene expression of miR-93 target genes in

glomeruli of AS *vs* WT (FC>1.5 or FC<-1.5, p-value<0.05, Dataset#1). Statistical analysis: differential expression determined using DESeq2; p<0.05 considered significant.

Supplementary Figure 4. Bulk-RNA seq on female AS glomeruli.

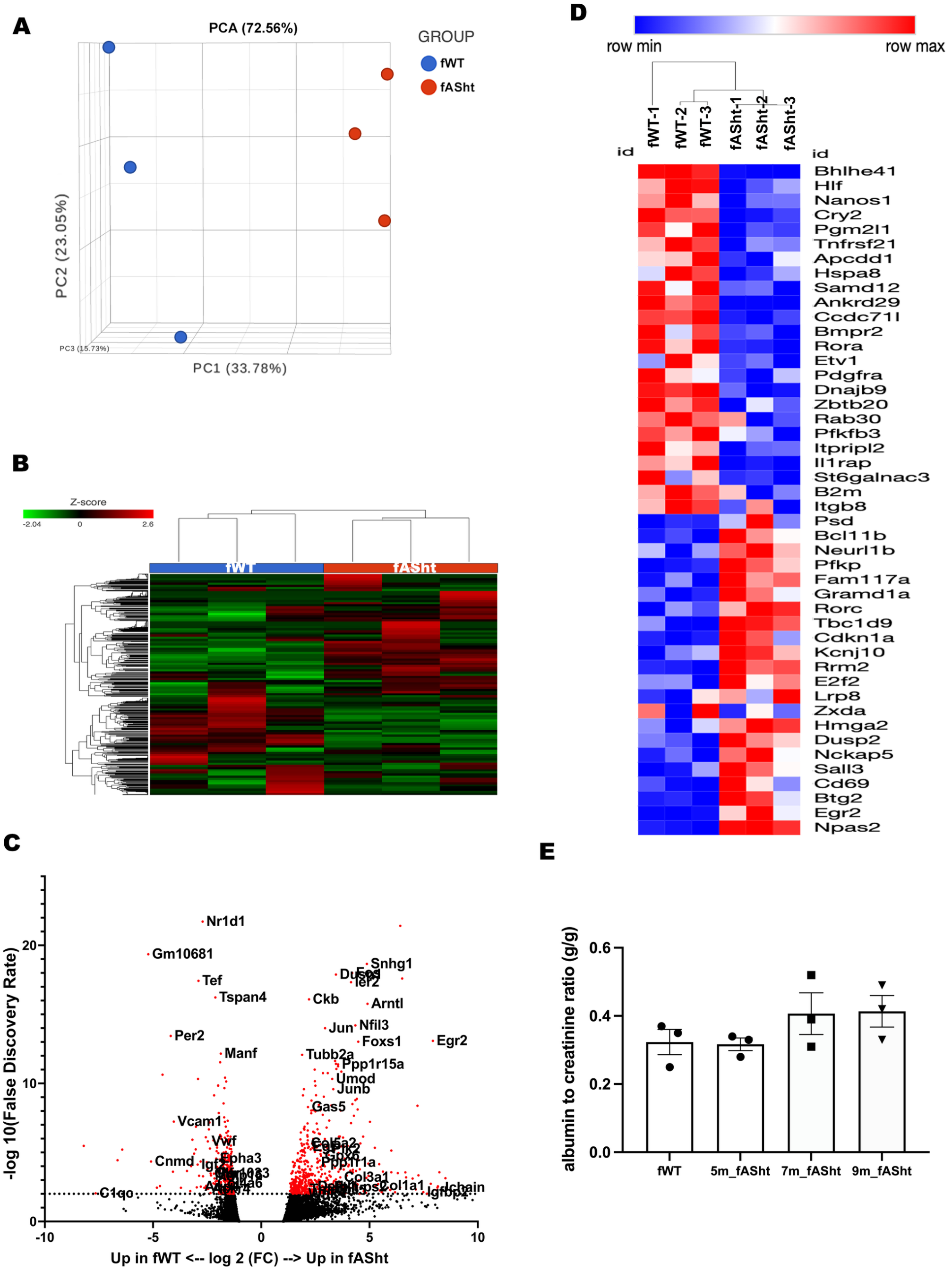

A. Principal component analysis (PCA) from the glomeruli of WT mice (blue, n=3, female, 7m old) and AS mice (heterozygous females) (red, n=3, AS hetero female, 7m old) showing the distribution of samples along PC1 (34% of the variance) and PC2 (23% of the variance). **B.** Hierarchical clustering heatmap representing DE genes in WT (blue group, left) and AS (red group, right),

showing a shift with AS disease. **C.** Volcano plot showing the DE genes in AS *vs* WT (FC>1.5 or FC<-1.5, p-value<0.05). **D.** Hierarchical clustering heatmap showing FC in gene expression of miR-93 target genes in glomeruli of AS *vs* WT (FC>1.5 or FC< -1.5, p-value < 0.05, Dataset#1). Statistical analysis: differential expression determined using DESeq2; p<0.05 considered significant.

Supplementary Figure 5: EV characterization and validation of miR-93 KD.

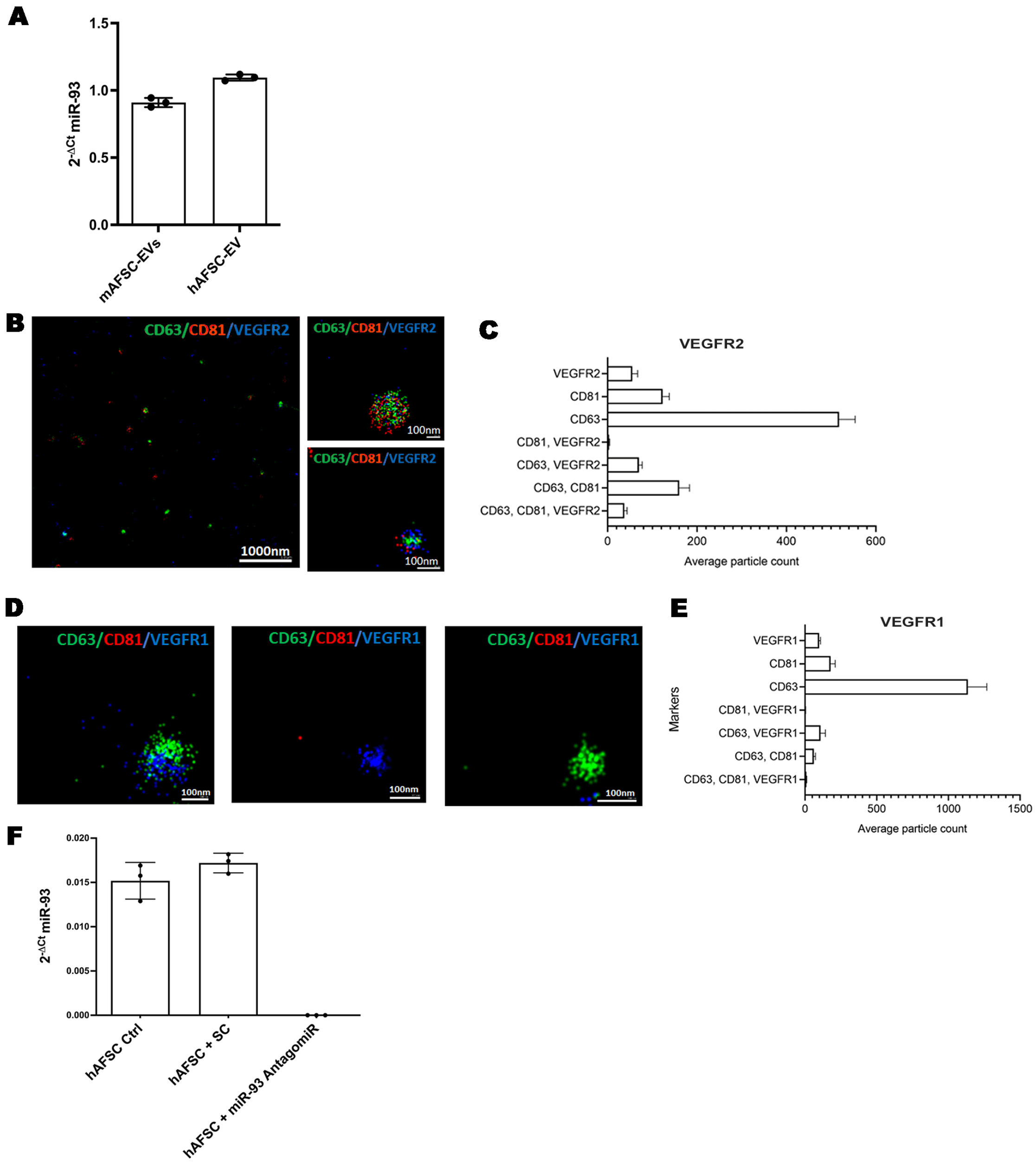

A. Graph showing miR-93 expression in EVs derived from mouse AFSC (passage 57) and human AFSC (passage 37). Small nuclear RNA U6 was used as a reference to calculate relative expression**. B-C.** Super-resolution (ONI) microscopy of the surface tetraspanin markers CD63, CD81 combined with VEGFR2 (B), with graph (C) representing the single, double, and triple

positive fractions. Scale bar:100nm **D-E**. Super-resolution (ONI) microscopy of the surface tetraspanin markers CD63, CD81 combined with VEGFR1 (D), with graph (E) representing the single and double positive fractions. Scale bar:100nm. **F.** Graph showing miR-93 expression in hAFSC in baseline, scramble control, and miR-93 KD hAFSC. Small nuclear RNA U6 was used as a reference to calculate relative expression, and all experiment was run in triplicate. hAFSC+SC: cells treated with miR-93 scramble control. Statistical analysis: One-way ANOVA with Uncorrected Fisher's LSD for expression data (A, F). Descriptive only for microscopy panels (B-E).

Supplementary Figure 6. EVs and KD_EVs in podocytes and mesangial cells.

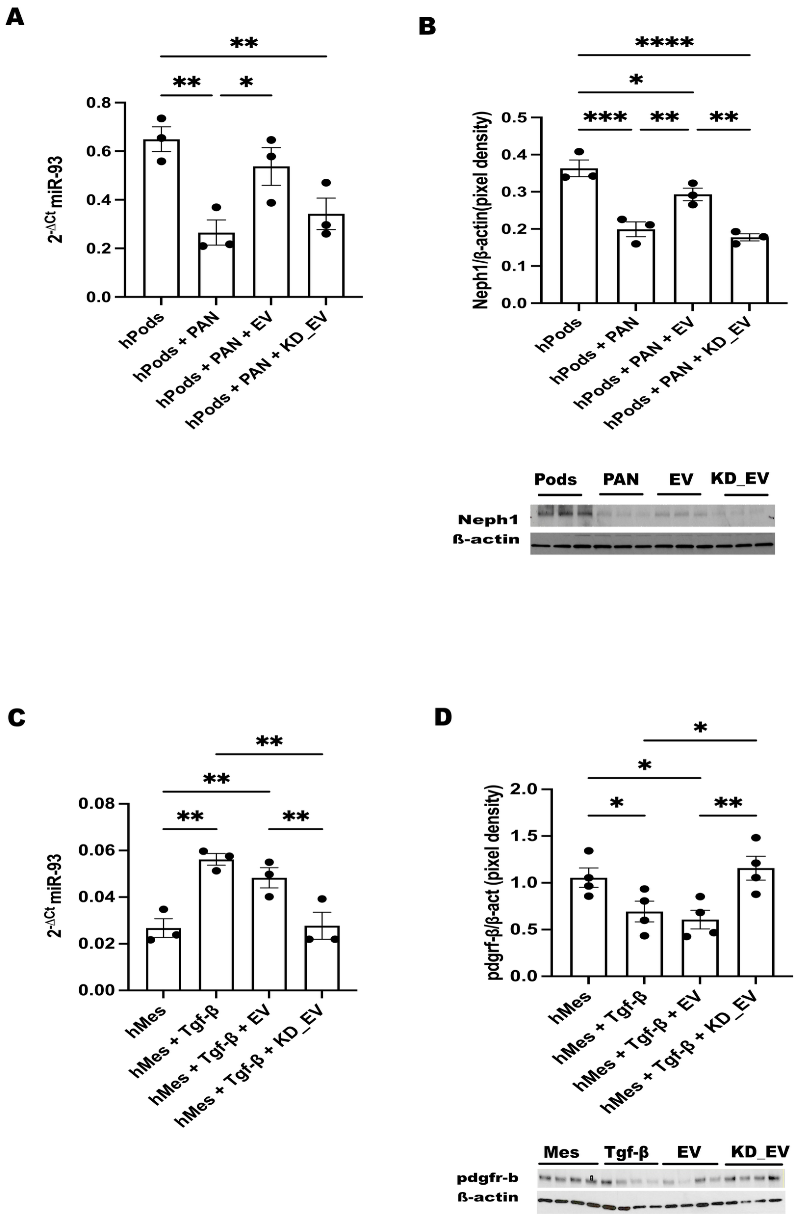

A.Graph showing miR-93 expression in podocytes at baseline, PAN-induced damage, PAN-induced damage plus EVs, and PAN-induced damage plus KD_EVs. Small nuclear RNA U6 was used as a reference to calculate relative expression. **B.** Graph showing densitometric analysis for Neph1 (110 kDa) in podocytes at baseline, PAN-induced damage, PAN-induced damage plus EVs, and PAN-induced damage plus KD_EVs. Quantification was measured based on ß-actin as housekeeping. WB bands are shown below the graph. **C.** Graph showing miR-93 expression in mesangial cells at baseline, TGF-ß-induced damage, TGF-ß-induced damage plus EVs, and TGFß-induced damage plus KD_EVs. Small nuclear RNA U6 was used as a reference to calculate relative expression**. D.** Graph showing densiometric analysis for PDGRF-b (151 kDa) in mesangial cells at baseline, TGF-ß-induced damage, TGF-ß-induced damage plus EVs, and TGFß-induced damage plus KD_EVs. Quantification was measured based on ß-actin as housekeeping. WB bands are shown below the graph. All experiments are repeated in triplicate. All values are reported as mean ±SEM. Statistical analysis: One-way ANOVA with Uncorrected Fisher's LSD. Significant values are indicated as: * ($p<0.05$), ** ($p<0.01$), *** ($p<0.001$), **** ($p<0.0001$).

Supplementary Figure 7. 10X Genomics workflow.

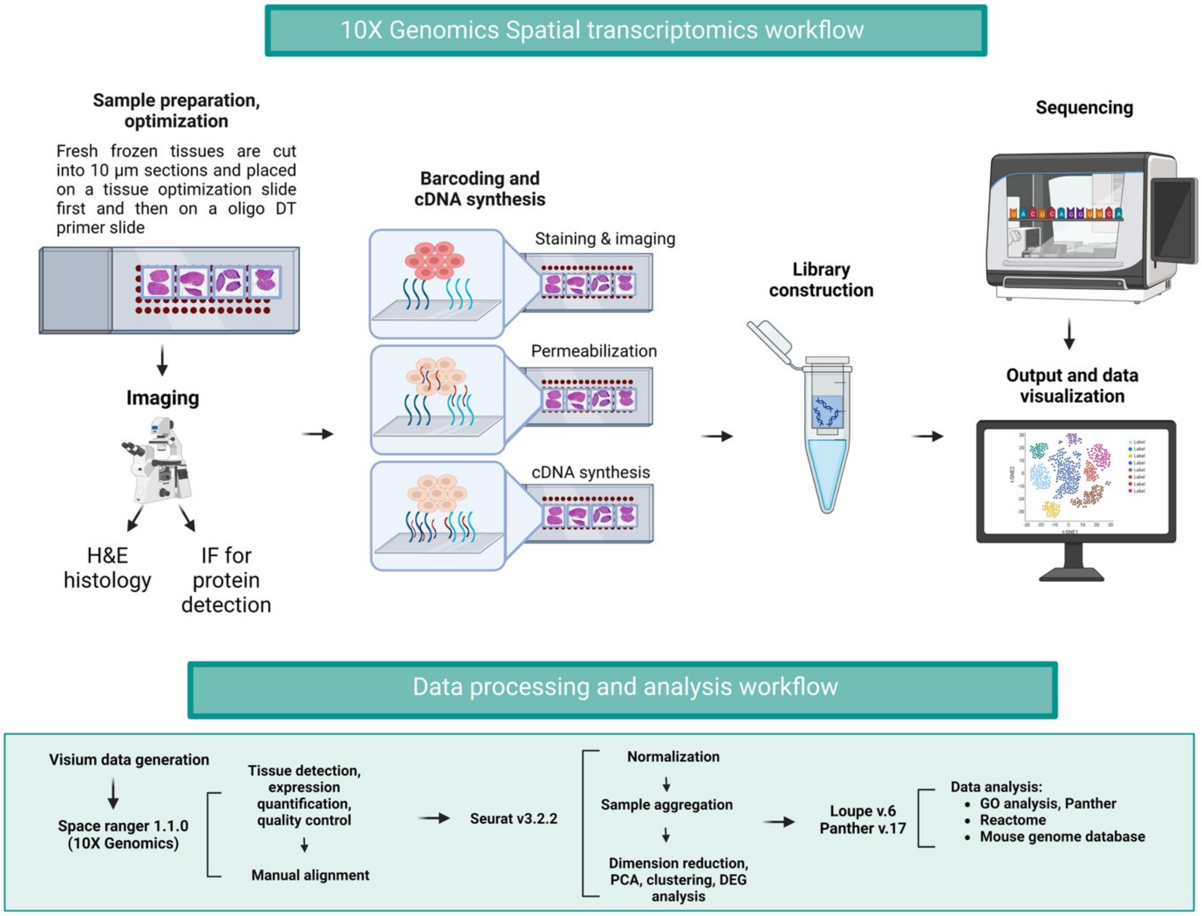

Schematic representation of the 10x Genomics Spatial transcriptomics workflow.

Supplementary Figure 8: Spatial maps of the kidney from WT, 2M old AS, 5M old AS, and treated AS mice.

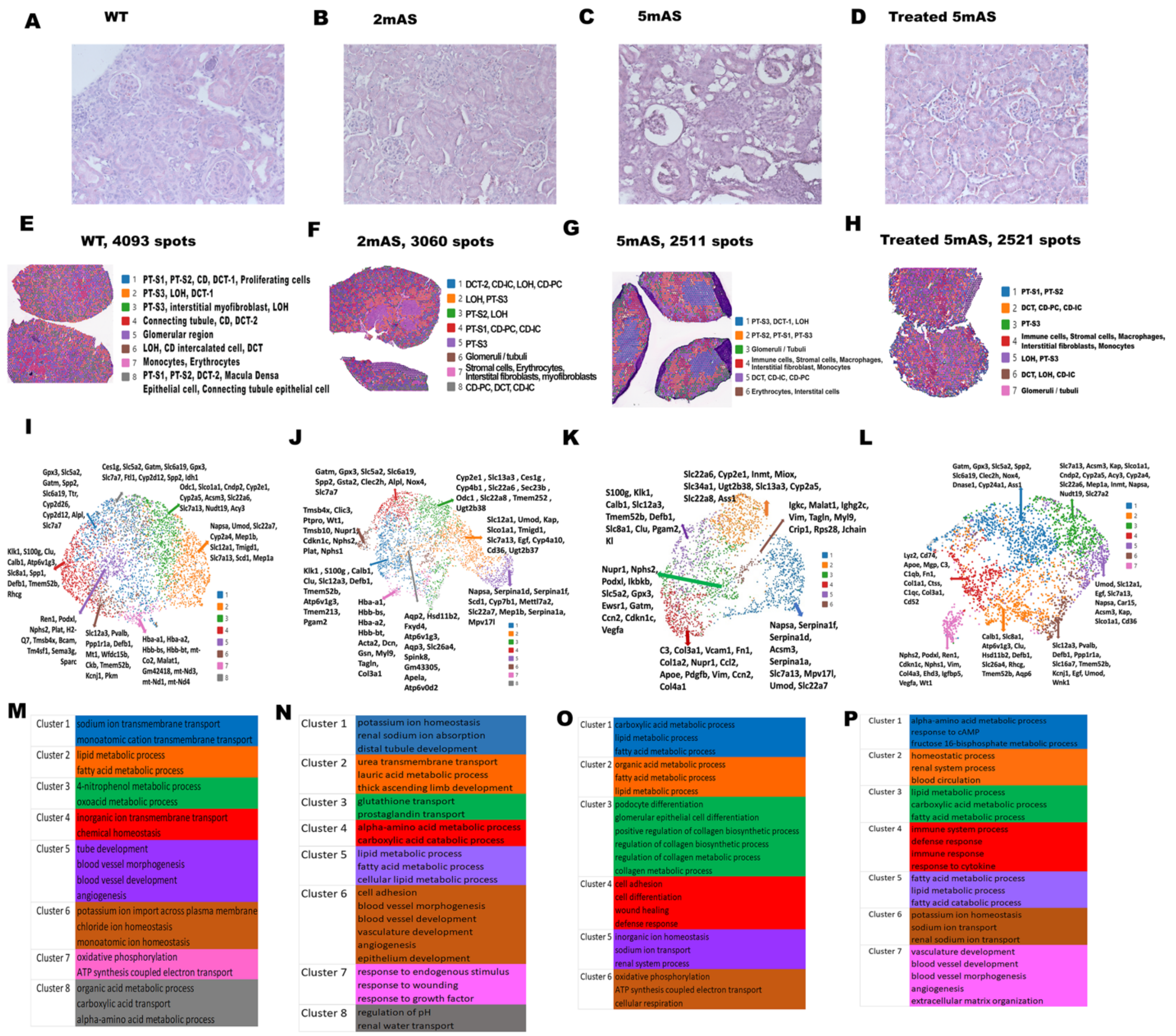

A-D. H&E staining of kidney sections used for the ST analysis of individual samples: WT 4m old (A), 2m old AS (B), 5m old AS (C), and treated 5m old AS (D) mice. Treated 5m old mice were injected with hAFSC-EVs at 2.5m old and sacrificed at 5m old. **E-H.** ST analysis of WT 4m old (E), 2m old AS (F), 5m old AS (F), and treated 5m old AS (H) by unsupervised clustering identified 8, 8, 6, and 7 clusters, respectively, as shown in the spatial map for each sample. **I-L**. UMAP of spots and specific genes identifying the clusters from individual samples of WT 4m old (i), 2m old AS (J), 5m old AS (K), and treated 5m old AS (L) are shown. **M-P.** Enriched Gene Ontology (GO) sets for biological processes obtained from the significantly upregulated genes in each cluster are shown in WT 4m old (M), 2m old AS (N), 5m old AS (O) and treated 5m old AS (P) mice. (log2FC >0.25 or log2FC<-0.25, p-value<0.05, Dataset#4-7). Statistical analysis: differential expression determined using DESeq2; adj. p<0.05 considered significant.

PT-S1: Proximal Tubules Segment 1; PT-S3: Proximal tubule Segment 2; PT-S3: Proximal Tubules Segment 3; DCT: Distal Convoluted Tubule; CCD: Cortical Collecting Duct; OMCD: Outer Medullary Collecting Duct; LOH: Loop of Henle; TAL: Thick Ascending Limb; DTL: Descending Thin Limb; ATL: Ascending Thin Limb; CD: Collecting Duct; IC: Intercalated Cells.

Supplementary Figure 9. Kidney cluster classification.

Cluster	Identification	Genes used to identify the clusters
1	Proximal tubules (S1)	Gatm, Slc6a19, Slc5a2, Gpx3, Spp2, Ass1, Alpl, Nox4, Slc22a8, Slc7a7, Miox, Pah
2	Proximal tubules (S3, S2)	Slco1a1, Cndp2, Inmt, Slc7a13, Slc22a6, Cyp2e1, Kap, Acy3, Nudt19, Slc27a2, Cyb5a, Azgp1, Amacr
3	Proximal tubules (S3), DCT, Proximal tubules (S1)	Napsa, Slc22a7, Mep1b, Slc7a13, Acsm3, Mpv17l, Umod, Mep1a, Crot, Kap, Tmigd1
4	Interstitial fibroblasts, Blood vessels, Immune cells	Lyz2, Mgp, Spp1, Cd74, C3, Apoe, Col3a1, Col1a2, Bgn, Col1a1, S100a6, Tmsb4x, Fn1, B2m, Vcam1
5	Distal convoluted tubules (DCT), Cortical collecting duct (CCD)	Slc12a3, Defb1, Calb1, Ppp1r1a, Tmem52b, Pvalb, Wnk1, Pgam2, Kcnj1, Slc16a7, Atp1a1, Pkm
6	Outer medullary collecting duct (OMCD), Cortical collecting duct (CCD)	Atp6v1g3, Calb1, Hsd11b2, Slc8a1, Rhcg, Tmem52b, Slc26a4, Spink8, Defb1, Tmem213, Scnn1b, Pgam2
7	Glomerular region	Podxl, Nphs2, Ren1, Cdkn1c, Angptl2, Nphs1, Ehd3, Igfbp5, Vim, Clic3, Col4a3, Vegfa, Wt1
8	Loop of Henle, Cortical collecting duct (CCD)	Aqp2, Umod, Slc12a1, Car15, Egf, Hsd11b2, Lgals3, Krt7, Mt1, Wfdc15b, Adgrg1, Mrps6, Ppp1r1a
9	Collecting duct (CD), Dendritic cells, monocytes, macrophages	Malat1, Sec23b, Cdk11b, Cfh, Eid2, Slc13a3, Pacs2, Proz, Zmynd10, Mark3

Table showing classification of each cluster from spatial transcriptomic analysis performed on integration of WT, 2m old AS, 5m old AS, and treated 5m old AS, also reporting the genes used to identify each cluster. Treated 5m old mice were injected with hAFSC-EVs at 2.5m old and sacrificed at 5m old. Dataset#8. Statistical analysis: differential expression determined using DESeq2; adj. $p<0.05$ considered significant.

Supplementary Figure 10. Gene ontology analysis of ST integrated samples.

A

AS progression

2mAS vs WT
cellular metabolic process
biosynthetic process
ATP synthesis coupled electron transport
ribonucleotide biosynthetic process
intrinsic apoptotic signaling pathway by p53 class mediator
Diabetic cardiomyopathy
Oxidative phosphorylation
SRP-dependent cotranslational protein targeting to membrane
L13a-mediated translational silencing of Ceruloplasmin expression

5mAS vs WT
extracellular matrix binding
extracellular matrix structural constituent
conferring tensile strength
collagen binding
fibronectin bindinh
programmed cell death
apoptotic process
cell death
response to wounding
T cell activation
chemotaxis
MAPK cascade
collagen-containing extracellular matrix
AGE-RAGE signaling pathway in diabetic complications
ECM-receptor interaction
ECM proteoglycans
collagen degradation

EV treatment versus AS

Treated 5mAS vs 2mAS
integrin binding
extracellular matrix binding
extracellular matrix structural constituent
conferring tensile strength
blood vessel morphogenesis
angiogenesis
apoptotic process
cell-matrix adhesion
collagen type IV trimer
collagen type I trimer
ECM-receptor interaction
extracellular matrix organization
collagen formation
ECM proteoglycans

Treated 5mAS vs 5mAS
tube development
blood vessel morphogenesis
tube morphogenesis
vasculature development
blood vessel development
angiogenesis
kidney development
renal system development
nephron development
animal organ development
glomerulus development
extracellular matrix organization
extracellular structure organization
glomerular epithelium development
wound healing
ERK1 and ERK2 cascade
podocyte development
ECM-receptor interaction
regulation of actin cytoskeleton

EV treatment versus WT

Treated 5mAS vs WT
actin binding
integrin binding
calcium ion binding
laminin binding
S100 protein binding
mRNA 3'-UTR binding
G protein-coupled receptor binding
tube morphogenesis
vasculature development
blood vessel development
animal organ development
regulation of cell migration
negative regulation of cellular process
blood vessel morphogenesis
response to wounding
angiogenesis
nephron development
wound healing
glomerulus development
renal system development
collagen metabolic process
glomerular basement membrane development
podocyte development
ECM-receptor interaction
extracellular matrix organization
ECM proteoglycans
collagen formation
collagen biosynthesis and modifying enzymes

B

Pathways ↑ in 5mAS vs WT
apoptotic process
programmed cell death
angiogenesis
inflammatory response
MAPK cascade
integrin-mediated signaling pathway

→

Pathways ↓ in treated 5mAS vs 5mAS
metabolic process
negative regulation of intrinsic apoptotic signaling pathway
negative regulation of programmed cell death
negative regulation of apoptotic process
regulation of programmed cell death
apoptotic process

Pathways ↓ in 5mAS vs WT
lipid metabolic process
acyl-CoA metabolic process
kidney development
positive regulation of insulin-like growth factor receptor signaling pathway
amino acid biosynthetic process
energy reserve metabolic process
NADP metabolic process
ATP synthesis coupled electron transport

→

Pathways ↑ in treated 5mAS vs 5mAS
blood vessel morphogenesis
tube development
blood vessel development
renal system development
angiogenesis
kidney development
glomerulus development
regulation of MAPK cascade

A. Enriched Gene ontology (GO) sets for biological processes obtained from the significantly upregulated genes in the glomerular spots of 2m old AS *vs* WT 4m old , 5m old AS *vs* WT 4m old, treated 5m old AS *vs* 2mAS, treated 5m old AS *vs* 5m old AS, treated 5m old AS *vs* WT 4m old and 5m old AS *vs* 2m old AS for glomerular region (cluster 7). Dataset#10. **B.** Enriched GO sets for biological processes based on significantly upregulated or downregulated genes in glomerular

spots of 5m old AS *vs* WT 4m old (left panels) and in downregulated or upregulated genes in glomerular spots of treated 5m old AS *vs* 5mAS mice. Dataset#10. Treated 5m old mice were injected with hAFSC-EVs at 2.5m old and sacrificed at 5m old. Statistical analysis: differential expression determined using DESeq2; adj. p<0.05 considered significant.

Supplementary Figure 11. Pathway analysis of the ST integrated glomerular cluster.

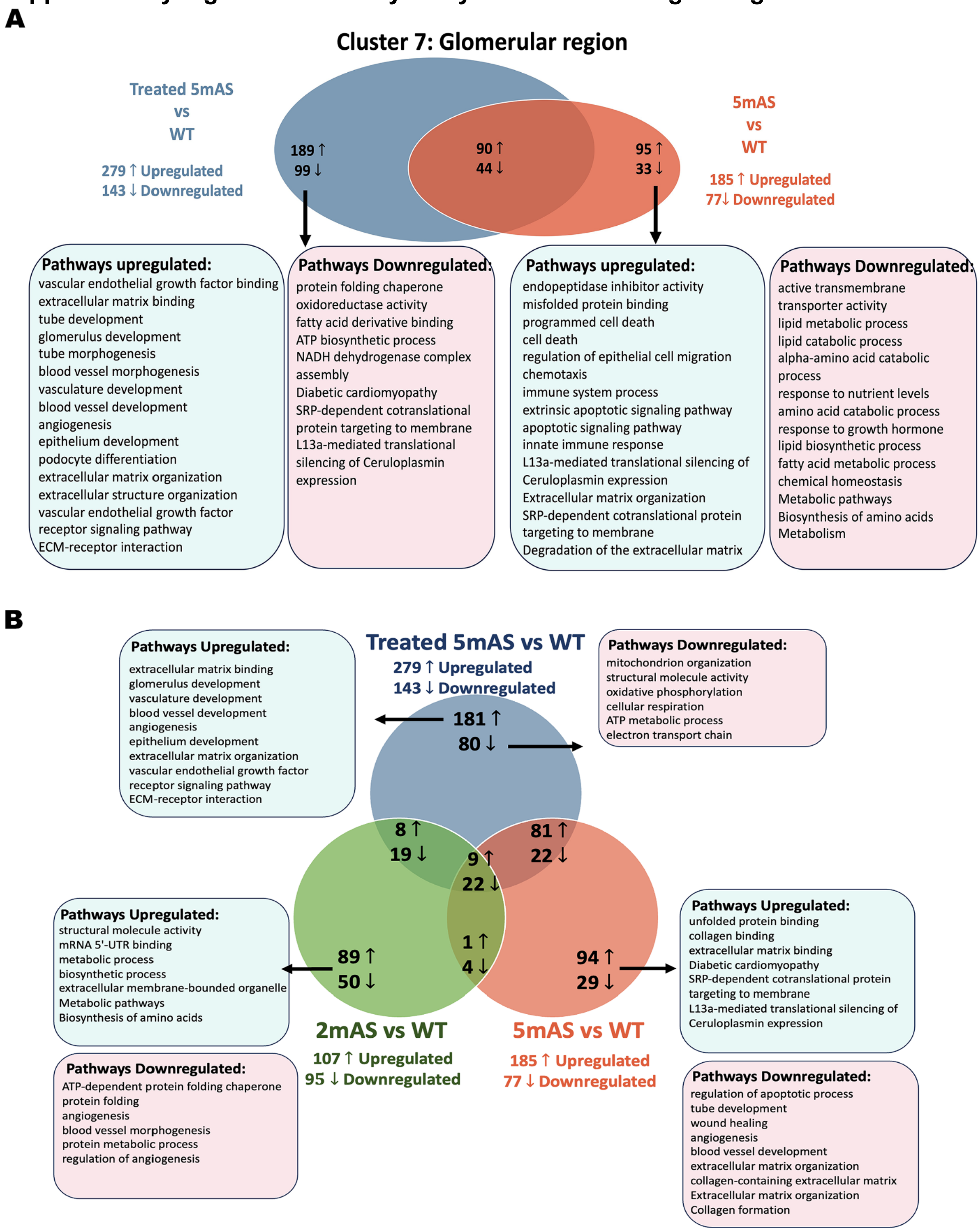

A. Venn diagram displaying the number of significantly upregulated genes in glomerular spots of treated 5m old AS *vs* WT (left panel) and 5m old AS *vs* WT (right panel). Highlighted below are significant upregulated Gene ontology sets (log2FC>0.25 or log2FC<-0.25, p-value<0.05). **B.** Venn diagram displaying the number of significantly upregulated genes in glomerular spots of treated 5m old AS *vs* WT (blue panel), 5m old AS *vs* WT (orange panel), and 2m old AS *vs* WT (green panel) (log2FC>0.25 or log2FC<-0.25, p-value<0.05, Dataset#13). Highlighted below are significant upregulated Gene Ontology sets. Treated 5m old mice were injected with hAFSC-EVs at 2.5m old and sacrificed at 5m old. Statistical analysis: differential expression determined using DESeq2; adj. p<0.05 considered significant.

Supplementary Figure 12. Glomerular Cluster classification.

Subcluster	Identification	Genes used to identify the clusters
1	Glomerular cells (Podocytes, GEC and mesangial cells), interstitial fibroblast	Nupr1, Nphs2, Lyz2, Sparc, Podxl, Vim, Spp1, Sema3g, Ccn2, Mgp, Col1a2, Cdkn1c, Col4a3, Igfbp5, Col4a1, Itgb1, Vegfa, Ehd3, Dock8, B2m, C3
2	Proximal tubules (S1, S2, S3)	Kap, Nudt19, Slc7a13, Dnase1, Cndp2, Acy3, Cyp4b1, Napsa, Slco1a1, Slc27a2, Mep1a, Inmt, Ttc36
3	Macula densa, juxtaglomerular cells, connecting tubule (CNT)	Klk1, Ren1, S100g, Slc12a3, Calb1, Clcnkb, Defb1, Ppp1r1a, Atp6v1g3, Car15, Rhcg, Tmem52b, Kcnj1, Pgam2, Egf
4	Immune cells (Dendritic cells, Macrophages, Monocytes)	Lyz2, Cd74, C3, Mgp, Apoe, Col3a1, Col1a1, C1qb, S100a6, Vcam1, Fn1, C1qa, Bgn, Col1a2, C1qc

Table showing classification of each subcluster from glomerular region (cluster 7) performed on integration of WT 4m old, 2m old AS, 5m old AS, and treated 5m old AS also reporting the genes used to identify each cluster. Dataset#14. Treated 5m old mice were injected with hAFSC-EVs at 2.5m old and sacrificed at 5m old. Statistical analysis: differential expression determined using DESeq2; adj. p<0.05 considered significant.

Supplementary Figure 13. FOV identification and cell annotation.

A

General and kidney specific patient data

Sample Name	Mutation	Age	Sex	sCreat. (mg/dL)	Protein/ Cr Ratio	Additional information
Reference	N/A	19	M	1.13	N/A	Transplant biopsy, no rejection, no IFTA, no vascular sclerosis; EM with normal GBM
#1	COL4A5 c.4853del (p.Gly1618Valfs*35); heterzygous	3	F	0.25	0.615	Atypical GBM characteristic of Alport
#2	COL4A5 c.1904 G>A (p.Gly635Asp); heterzygous	36	F	0.7	2.482	Atypical GBM characteristic of Alport; low grade IgAN, minimal arteriosclerosis/arteriolosclerosis, patchy acute tubular injury
#6	COL4A5 c.1001 G>C (Gly334Ala)	42	M	1.0	1.170	Atypical GBM characteristic of Alport; arteriolosclerosis

B

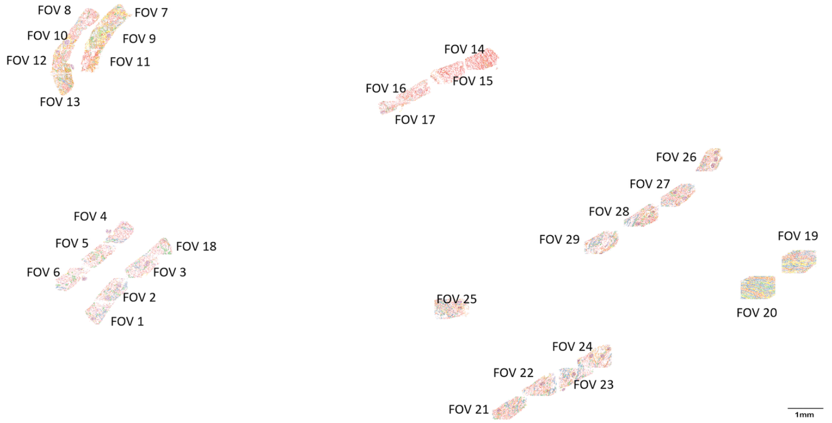

C

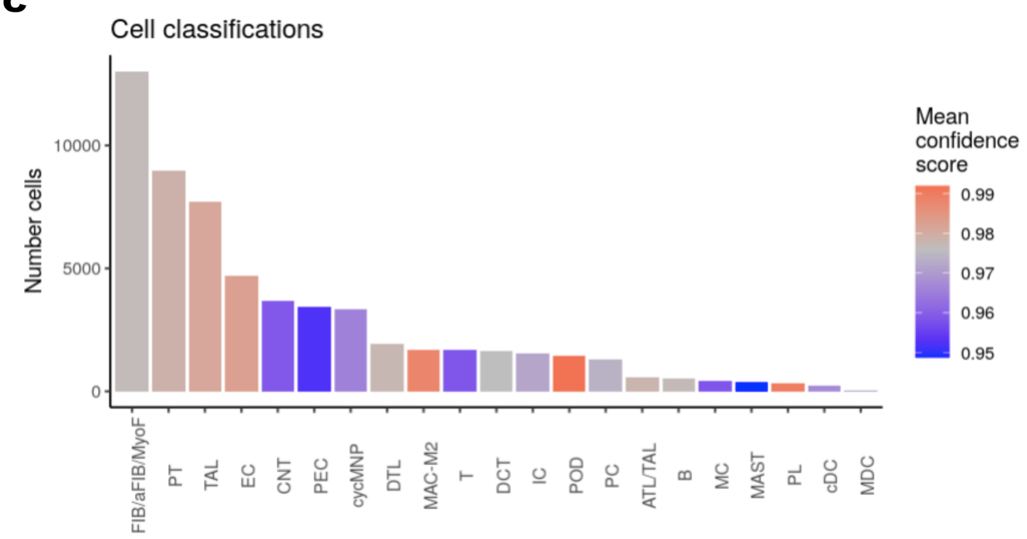

A. Table showing clinical, genetic and histopathology information of the kidnet biopsies analyzed. **B.** Visualization of the 29 FOVs from each biopsy. Patient genotypes are listed in Fig. 6B. **C**. Bar graph showing quantification of cell numbers and mean confidence scores for each classified cell types. Data were aggregated from all 29 FOVs.

Supplementary Figure 14. Cell annotation per FOV and cell segmentation.

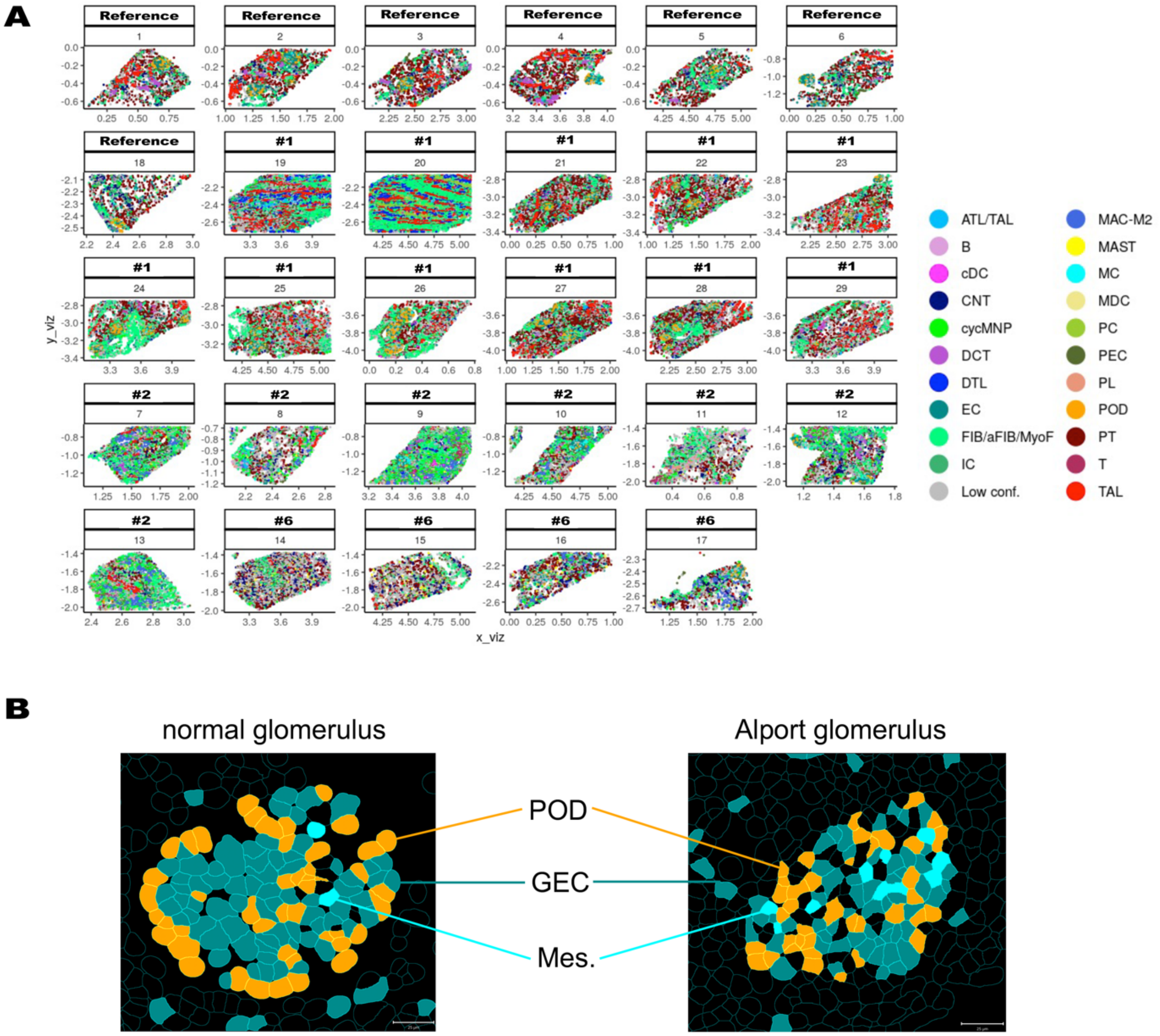

A. Visualization of individual FOVs from each biopsy showing the spatial distribution of the 21 cell types as indicated by color codes for each FOV analyzed. **B.** Representative glomerulus from reference (non-diseased patient, left panel) and AS from biopsy #1 (right panel) showing cell segmentation and identification of podocytes (orange), GEC (blue), mesangial cells (light blue). Cells that failed to pass QC thresholds are represented in black. Scale bar, 25um. Images were obtained with Napari 4.0.17 software.

Supplementary Figure 15. Distribution of podocytes, glomerular endothelial cells (GEC), mesangial cells and parietal epithelial cells (PEC) in each FOV.

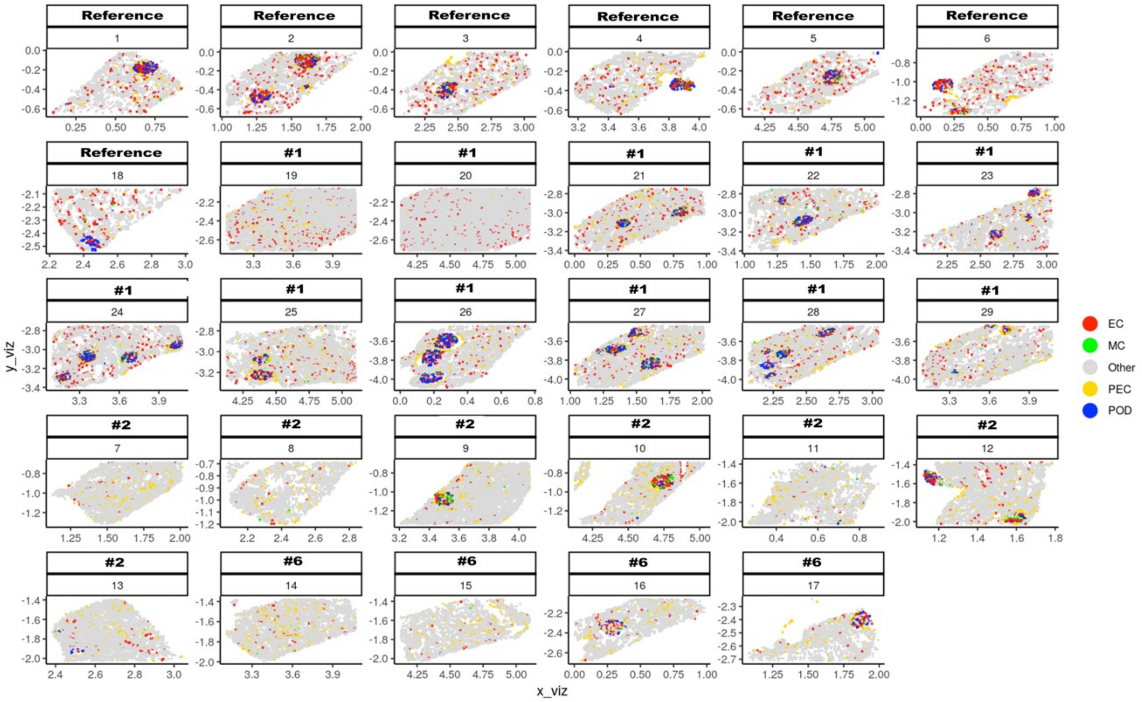

Visualization of individual FOVs from each biopsy showing the distribution of the podocytes, glomerular endothelial cells (GEC), mesangial cells and parietal epithelial cells (PEC) as indicated by color codes for each FOV analyzed.

Supplementary Methods

AFSC culture, EV isolation, EV characterization, TEM, and Super Resolution Microscopy.

AFSC were isolated and cultured from total human amniotic fluid as published (1-4). Briefly, cells were selected for c-kit expression by magnetic activated cell sorting (MACS) and clonal lines were generated and cultured in non-treated culture flasks (Corning, C#430825) with α-MEM Medium (Gibco, C# 12571063) supplemented with 20% Chang Medium B (Irvine Scientific, C#C100) and 2% Chang Medium C (Irvine Scientific, C#C106-14 mL), 20% Fetal Bovine Serum (GIBCO, C#16141), 1% L-Glutamine (Gibco, C#25030081), and 1% Pen-strep (Gibco, C#15140122). All the experiments were performed using our established and validated clonal line.

For EV isolation, hAFSCs were cultured in medium without serum to avoid EV bovine contamination for 24hrs. Medium was then collected and centrifuged at 6,000g for 30′ to remove cellular debris, and EVs were isolated by ultracentrifugation at 100,000g (Optima L-100K ultracentrifuge; Beckman Coulter) for 2hrs at 4°C; pellets were resuspended in PBS and stored at −80°C until use. The size and distribution of the EVs were analyzed using a NanoSight NS300 instrument (Malvern Panalytical, UK) equipped with a green 532nm laser and a sCMOS camera. The samples were diluted to obtain the optimal detection concentration of 10^8 particles/ml; an automated syringe pump was used to achieve a constant flow speed of 25, and five videos were captured using camera level 14. The data were analyzed using NTA software 4.3 with a detection threshold of 7 and adjusted by the dilution factor.

For TEM, $5.8x10^8$ EVs suspended in 25μl of PBS were placed on a 400-mesh holey film grid; after staining with 2% uranyl acetate for 2’, the sample was observed with a Tecnai G2 transmission electron microscope (FEI Company) operating at 100 kV. Images were captured with a Veleta digital camera (Olympus Soft Imaging System).

Super-resolution microscopy pictures of EVs were obtained using a temperature-controlled Nanoimager S Mark II microscope from ONI (Oxford Nanoimaging) equipped with a 100x, 1.4NA oil immersion objective, an XYZ closed-loop piezo 736 stage, and 405 nm/150 mW, 473 nm/1 W, 560 nm/1 W, 640 nm/1 W lasers and dual or triple emission channels split at 640 / and 555 nm. EV profiler Kit (ONI) was used to perform the experiments following the manufacturer's protocol. The Kit contains fluorescent antibodies anti CD9-488, CD63-568, and CD81-647, and the imaging buffer. In selected experiments, 2.5μg of purified mouse anti-VEGFR1 and anti-VEGFR2 antibodies (Santa Cruz) were conjugated with Alexa Fluor 647 dye and Alexa Fluor 555 dye, using the Apex Antibody Labelling Kit (Invitrogen) according to the manufacturer's protocol. One μl of each antibody was added to the blocking buffer at a final dilution of 1:10. Samples were incubated in the dark at 4°C. The day after, samples were washed twice with PBS, and ONI BCubed Imaging Buffer (Alfatest) was added for amplification of the EV signaling. Before each imaging session, bead slide calibration was performed to align the channels, to achieve a channel mapping precision smaller than 12 nm. Images were taken in dSTORM mode using 30% laser power for the 647 nm, 50% laser power for the 488 nm laser, and 30% for the 555 channel. Two-channel (647 and 555) dSTORM data (5000 frames per channel) or three-channels (2000 frames per channel) (647, 555 and 488) were acquired sequentially at 30Hz in total reflection fluorescence (TIRF) mode. Single-molecule data was filtered using NimOS software (v.1.18.3, ONI) based on the point spread function shape, photon count and localization precision to minimize background noise and remove low-precision and non-specific colocalization. Data were processed with the Collaborative Discovery (CODI) online analysis platform www.alto.codi.bio from ONI and the drift correction pipeline version 0.2.3 was used.

Isolation and culture of human podocytes, GEC, and mesangial cells.

Isolation of podocytes, GEC, and mesangial cells was performed as previously published (5-6), after mechanical and chemical digestion of the kidney cortex and glomerular isolation. Briefly, the

cortex was minced and digested in 125 U/ml collagenase I (Worthington, LS004197) in RPMI1640 at 37°C for 30' and filtered three times in 100-μm cell strainers (Corning, C# 352360) and once on the 40-μm cell strainer (Corning, C# 352340). The isolated glomeruli were trypsinized (Trypsin-EDTA; Gibco, C# 25200072) using 0.25% trypsin-EDTA for 5'. Glomerular endothelial cells were positively selected using CD31 (Miltenyi Biotech, C#130-091-935) by magnetic separation using MS separation columns. Mesangial cells were positively selected for CD140b (Miltenyi Biotech, C#130-118-985). Podocytes were positively selected for nephrin (Abcam, C# ab136894) using anti-rabbit IgG Microbeads (Miltenyi Biotech, C#130-048-602) using the manufacturer's instructions. Once sorted, the podocytes are seeded onto onto tissue culture plates coated with collagen I (Corning, C#354236) in VRADD medium (RPMI-1640 (Gibco, C#11875093) supplemented with 5% FBS (Gibco, C#26140079), 1% antibiotic (Gibco, C#15070063), 1,25(OH)2D3 [100 nM, cholecalciferol] (Sigma, C#C9756), all trans retinoic acid (ATRA) [1 μM], dexamethasone [100 nM] (Sigma, C#D4902). GEC are plated onto gelatin (Cell Biologics, C# 6950) in human endothelial cell medium (Cell Biologics, C# H1168) supplemented with 0.1% VEGF, 0.1% heparin, 0.1% EGF, 0.1% FGF, 0.1% hydrocortisone, 1% L-glutamine, 2% endothelial cell supplement, 10% FBS and 1% antibiotic-antimycotic solution (CellBiologics, C#6920). Mesangial cells are cultured in human mesangial medium (ScienCell Research Laboratories, C#4210) supplemented with 1% antibiotic (C#), 5% FBS (C#), Mesangial Cell Growth Supplement (ScienCell Research Laboratories, C#4252) and cultured for no more than 5 passages.

Mouse glomeruli isolation and isolation of mouse podocytes, GEC, and mesangial cells. For each mouse, the kidneys were harvested, the capsule and medulla removed. The tissue was mechanically minced for 5' and digested at 37°C in a collagenase type I solution (300U/mL, 1mg/ml; Worthington Biochemical corporation, C#LS004217) in RPMI (ThermoFisher Scientific, #11875093) for 40'. Tubes were shaken at regular intervals to help tissue digestion and finally

vortexed before further processing. Tissue homogenates were filtered twice through 100-μm Falcon cell strainers (Fisher Scientific, C#352360). Each strainer was rinsed thoroughly with PBS 1X containing1% BSA (Jackson ImmunoResearch, C#001-000-162) to prevent clumping. Glomeruli were collected from the top of the second cell strainer by inverting the strainer on a clean 50 ml tube and rinsing with a solution 1% BSA in PBS, and inspected under a microscope to determine purity; if needed, a second filter process through a 100-μm cell strainer followed by 40μm cell strainer was performed. Glomeruli were pelleted by centrifugation at 2000 rpm for 10' and were ready for analysis. To obtain mouse glomerular cells, glomeruli are further digested with 0.6% collagenase IV (Worthington, C#LS004188) for 15′ at 37 °C to obtain single cells. Podocytes and GEC were FACS sorted based on antibody staining for nephrin (Abcam, C#ab136894, 3ug/ml) and VE-cadherin (Abcam, C#ab33168, 1.5ug/ml); mesangial cells were considered as the negative fraction of the nephrin-VE-cadherin positive selection. Primary antibodies were conjugated with either Zenon kit fluorescent dye (Invitrogen, C#Z25302 and C#Z25055) at 1/2000 dilution for 5' at 25°C followed by 5' incubation with Zenon blocking solution at 25°C. The cells were then incubated with primary antibodies for 40 min at 4°C and sorted using a FACSAria III Cell Sorting System (BD Biosciences). Gating strategy is shown in Fig.S1. Isotype anti-rabbit IgG antibody was used as a control, and unlabeled cells were used as a negative control.

Western blot analysis.

Total protein from cells were collected in RIPA buffer (Santa Cruz, C#sc-24948) with protease and phosphatase inhibitors and stored at −80°C (ThermoFischer Scientific, C#89901). Samples were prepared by adding lane marker reducing sample buffer (Thermofisher Scientific, C#39000), heated to 95°C for 5' and separated on a 4-20% Tris-Glycine gel (Biorad, C#5678083) and transferred using the Transblot turbo system with PVDF transfer packs (Biorad, C#1704157). After soaking in methanol for 10', membranes were washed and blocked in 5% Milk (Santa Cruz Biotechnology, C#sc-2324 for 1hr and probed with primary antibodies overnight at 4°C (list of

antibodies and specific concentrations below). HRP-conjugated secondary anti-rabbit antibodies (ThermoFischer Scientific, C#31460) were applied it 1:20,000 ratio. Antigens were detected using the SuperSignal West Femto Maximum Sensitivity Substrate (Thermofisher Scientific, C#34096), impressed on Amersham Hyperfilm MP (Cytiva, C#28906842). Data from 3 independent experiments were quantified by densitometry (all measurements were normalized against their corresponding housekeeping gene, β-actin; for EVs, WB normalization was evaluated by adding the same amount of protein/group). The list of all antibodies and concentrations used for WB is reported in Table 1 below.

Primary Antibody	Company	Catalog #	Concentration
VEGF	Abcam	ab9570	WB 1:2000
Fibronectin	Abcam	ab2413	WB 1:1000
VEGFR1	Abcam	ab32152	WB 1:500
VEGFR2	R&D	AF357-SP	WB 1:500
p-VEGFR2	Abcam	ab5673	WB 1:100
PDGRF-b	Abcam	ab32570	WB 1:1000
WT1	Abcam	ab89901	WB 1:250
Neph1	Santa Cruz	SC-373181	WB 1:500
β-actin	Gentex	109639	WB: 1:1000

Table 1. List of the antibodies and concentrations used for WB experiments.

RNAScope in-situ hybridization.

miRNAScope (Advanced Cell Diagnostics) was performed on sections from paraffinized kidney biopsies following manufacturer's protocols. Briefly, slides were baked for 1h at 60°C, deparaffinized, dehydrated, and incubated in 10% Neutral Buffered Formalin (VWR, C#

89370094) O/N at RT. Slides were washed in DI H2O for 2', warmed for 5' at 60°C, incubated for 10' at RT with RNAscope H2O2 (ACD Bio, C#322335), rinsed and for 15' at 100°C in 1X Target Retrieval Reagent (ACD Bio, C#322000). After washing, RNAscope Protease III (ACD Bio, C#322337) was added for 20' at 40°C; slide were washed and treated with miRNAscope HD Detection ReagentsRED (ACD Bio, C#324510). Slides with miRNAScope positive control (ACD, C#727871-S1) and negative control (ACD Bio, C#727881-S1) were incubated for 1hr while slides with miRNAscope human miR-93 probe (ACD Bio, C#895271-S1) for 2hrs at 40°C. After rinsing, slides were incubated with Amp 1 (ACD Bio, C#324511) for 30' at 40°C, followed by Amp 2 (ACD Bio, C#324512) for 15' at 40°C, Amp 3 (ACD Bio, C#324513) for 30' at 40°C, Amp 4 (ACD Bio, C#324514) for 15' at 40°C, Amp 5 (ACD Bio, C#324515) for 1hr at RT, Amp 6 (ACD Bio, C#324516) for 30' at RT. After washing, slides were incubated with Fast Red at 1:60 of Red B (ACD Bio, C#324518) and Red A (ACD Bio, C#324517) in the dark for 10' at RT and blocked in 2% BSA (Jackson ImmunoResearch, C#001-000-162) O/N at 4°C. The slides were then stained for EHD3 (Sigma-Aldrich, C#HPA049986) for 2hrs at RT followed by washes in 1X PBS and incubation with anti-Ab Alexa Fluor 488 (Thermofisher, C#A-21206) for 30' at RT, followed by DAPI (BD Pharmingen, C#564907) for 15' at RT, and mounted with Prolong Gold antifade mountant (Thermofisher, C#P36930). Pictures were acquired on confocal microscopy (Zeiss 710 microscope) and processed using the ZEN10 software.

Co-immunoprecipitation assay.

To evaluate VEGF/VEGFR1 binding in hAFSC-EVs and hAFSC-EVs $^{miR\text{-}93\text{-}/\text{-}}$, EVs were resuspended in 1ml PBS and treated with VEGF (Thermofisher Scientific, C#Phc93943, 100ug/ml). Immunoprecipitation for VEGFR1 was performed using VEGFR1 antibody and Protein G-agarose conjugate (Santa Cruz, C#sc-2003) overnight at 4°C. Immunoprecipitates were collected by centrifugation at 1,000xg for 5′ at 4°C, washed with PBS, and resuspended in electrophoresis sample buffer, denatured, and run under reducing conditions as previously

described (1). The immunoblots were then probed for VEGF detection using VEGF antibody (Abcam, C#ab46154, 1:1000 overnight at 4°C), followed by incubation with VeriBlot-HRP conjugated secondary antibody and detected by chemiluminescent substrate as described above.

Glomerular endothelium on a chip (GEOAC) and cell seeding.

GEOACs were generated using the OrganoPlate™ three-lane chip (Mimetas BV, the Netherlands, as published (5-6)). 3.0 µl of gel composed of 4 mg/ml Collagen I (AMSbio Cultrex 3D Collagen I Rat Tail, 5 mg/ml, c#3447-020-01), 100 mM HEPES (Life Technologies, c#15630–122) and 3.7 mg/ml NaHCO3 (Sigma, c# S5761) was dispensed in the gel inlet (middle) and incubated 2030' at 37°C. Human GECs were trypsinized by using 0.05% trypsin-EDTA (Gibco,c#LS25300062), and pelleted by centrifugation at 300g for 5 mins. 30,000 GEC were labeled with Cell Tracker Deep Red following manufacturing instruction (Invitrogen, c#c34565) and seeded into the inlet of the Channel C at a density of 1.5×10^7 cells/ml. Subsequently, the OrganoPlate™ was placed on its side at an angle for 30' at 37°C to allow the cells to sediment against the collagen I. This was followed by the addition of 50 µl of GEC medium to both the inlet and outlet of the top channel (Channel C) and the OrganoPlate™ was incubated on its side overnight at 37°C to complete the cell attachment. The OrganoPlate™ was placed horizontally in the incubator (37°C, 5% CO2) on an interval rocker switching inclination every 10', allowing bi-directional flow that enables media recirculation within each GEOAC. The media was changed every 2 days. Formation of a vascular channel was confirmed visually at day 5. At day 6, GEOAC were exposed for 6hrs to 400ng/ml VEGF (ThermoFisher, c#Phc9394) with or without EV or KD_EV (1:10,000 cell:EVs). GEOACs in regular media were used as control. After 6hrs, an albumin permeability assay was performed as previously described [5-6]. Briefly, media was collected from the bottom inlet and outlet (Channel F) to which PBS was added. Then, media from the top inlet and outlet were collected. 50 µl albumin-FITC (Millipore Sigma, c#A9771) was added to the top inlet and outlet. The GEOACs were imaged at 5' and 60',

during which the plates continued to incubate at 37°C. At 60’, media was collected from the bottom inlet and outlet. Absorbance was measured using the Perkin Elmer Victor 3 plate reader using Wallac 1420 workstation software (fluorescein 485/535, 0.1s). Albumin leakage was quantified as the fluorescent signal of the filtrate collected from the bottom channel.

In vivo study.

For in vivo experiments, a total of n=43 mice were used: non-injected control Alport mice (n = 14), Alport mice injected with hAFSC-EVs (n = 15) and Alport mice injected with hAFSC-EVs$^{miR\text{-}93\ -/-}$ (n=14). An extra 5 mice, WT C57, were used as a control. Mice were injected with 2.8×10^{10} EVs (100 uL) through the left ventricle at 2.5m, before the onset of proteinuria. Urine was collected every 2wks and renal function was assessed as previously described (1). Both urinary albumin concentration (Mouse Albumin ELISA Kit, C#E-90AL) and urinary creatinine concentration (BioAssay Systems, C#DICT-500) were determined by ELISA. Urinary albumin levels are presented as the albuminto-creatinine ratio (ACR; g/g). Blood was collected by facial vein as published (1). Blood samples (30 µL) were collected into plasma separation tubes with lithium heparin (BD, C#365987). Serum creatinine levels were measured by colorimetric assay (BioAssay Systems, C#DICT-500).

EV miRNA sequencing analysis.

Total RNA was isolated using exoRNeasy Midi Kit (Qiagen, C#77144). RNA integrity was checked by Agilent Bioanalyzer 2100. Library for small RNA-Seq was prepared according to Qiagen QIAseq miRNA library kit using 18.5-31.0 ng (5ul) total RNAs as input. Final library quality and quantity were analyzed by Agilent Technologies 4200 Tape station and Life Technologies Qubit3.0 Fluorometer. Single-end 75 bp reads were sequenced on Illumina Novaseq S4 (Illumnia Inc.). Reads are first processed by trimming off the 3’ adapter and low-quality bases using cutadapt

(cutadapt.readthedocs.io/en/stable/guide.html). Reads with no adapter sequence are tallied (no_adapter_reads). Following trimming, the insert sequences and UMI sequences are identified. Reads with less than 16 bp insert sequences (too_short_reads) or less than 10 bp UMI sequences (UMI_defective_reads) were discarded. The insert sequences were mapped to miRNA database (miRBase V21) using the bowtie. TMM normalization and differential expression analysis were carried out using the EdgeR package from Bioconductor. The cutoff of differential miRNA was a FC>1.5 or FC<-1.5 and p-value<0.05. IPA (Ingenuity Pathway Analysis) was used for the miRNA target genes analysis.

EV Proteomics analysis.

All samples (2.8x 10^10 EVs/sample) were kept on ice through the preparation process. Ultrapure water was used to bring samples to a volume of 200 µL, and 1000 µL of ice-cold 2:1 chloroform:methanol solution was also added. The samples were vortexed for 1 minute, placed on ice for 5' and vortexed again for 1'. Samples were centrifuged at 10k x g for 10' at 4°C. Following centrifugation, the polar (top) layer was removed, leaving a small layer behind since there wasn't a visible protein interphase layer. Using another Pasteur pipet, the non-polar (lower) phase was removed, and 1 mL of methanol was placed in the sample tube where the Pasteur pipet could be swirled and swished to remove any potential protein that had clung to the glass pipet. The samples were once again centrifuged as before to pellet the precipitated protein, the methanol was gently poured off, and the tube was drained upside down for 5 minutes before storing the samples in the freezer until further processing could occur. Proteins were solubilized in 50 mM NH4HCO3, 8 M urea, and DTT was added to reach 5 mM. Samples were incubated 30 min at 37°C with shaking at 800 rpm, iodoacetamide was added to reach a final concentration of 400 mM followed by a 60 min incubation at 37 °C with shaking at 800 rpm. After a 10-fold dilution in 50 mM NH4HCO3, samples were digested overnight with trypsin (1:50 trypsin:protein ratio) and 1 mM CaCl2. Peptides from copurified proteins were desalted using a C18 ultramicrospin column (3–30 µg

capacity; Nest Group), and peptides from AT2 samples were desalted with C18 solid phase extraction (Strata, 50 mg/mL, Phenomenex). Samples were concentrated and then evaporated using a speedvac to a volume of 25 µL. 5 µL of the 0.1 µg/µL digested peptides were analyzed by reverse-phase LC-MS/MS using a Dionex Ultimate 3000 RSLC nanopump (ThermoFisher Scientific) coupled with a Lumos Orbitrap mass spectrometer (ThermoFisher Scientific). The LC was configured to first load the sample on a solid-phase extraction (SPE) column (150 µm inner diameter, packed with 5 µm Jupiter C18 material (Phenomenex) followed by separation over a 120-minute gradient on an analytical column (25 cm, 75 µm inner diameter, packed with 1.7 µm BEH C18 material (Waters). Effluents were analyzed with the Orbitrap mass spectrometer operated in the data-dependent acquisition mode, first mass spectrometry survey was performed at a resolution of 60,000 with an automatic gain control (AGC) target at $4x10^5$. The top 12 ions with a charge comprised between 2 and 6 from survey scans selected for high-energy dissociation. An isolation window of 0.7 Da was used for the isolation of ions, and a collision energy of 30% was used for high-energy collisional dissociation with an automatic gain control setting of $3x10^6$ ions. MS/MS scans were acquired at a resolution of 50,000 with an AGC setting of $1x10^5$ ions and a maximum injection time of 105 ms. Mass spectra were recorded for 125 min with a dynamic exclusion window of 45 seconds.Raw MS data were analyzed using MaxQuant 1.6.0.16 against Uniprot nonredundant Homo sapiens database (20,361 sequences downloaded 03/07/2022) and contaminants. Two unique peptides were required, and False Discovery Rate (FDR) was set to 1% at the spectral, peptide, and protein levels. Statistical analyses were performed using the house made R package RomicsProcessor v1.0.0 (https://github. com/PNNL-Comp-Mass-Spec/RomicsProcessor). Briefly, the data were log2-transformed and median normalized. ANOVA, Student's T.test and presence-absence binomial general linear model (GLM) tests were performed. For the protein modulated in abundance or differentially present in groups (adj_p-value<0.05, log2FC>0 or log2FC<0), enrichment analyses were performed using a modified Fisher's exact test (EASE scores (7)). The raw mass spectrometry file and MaxQuant

search results were deposited on MassIVE, the dataset identifier is MSV000096685 (ftp://massive.ucsd.edu/v07/MSV000096685).

Bulk-RNA sequencing analysis of mouse glomeruli.

Total RNA was isolated using RNeasy plus Mini Kit (Qiagen, C#74134). RNA integrity was checked by Agilent Bioanalyzer 2100. Library for RNA-Seq was prepared according to KAPA Stranded mRNA Hyper prep polyA selected kit with 201-300 bp insert size (KAPA Biosystems) using 250ng total RNAs as input. Final library quality and quantity were analyzed by Agilent Technologies 4200Tape station and Qubit 3.0 (Thermo Fisher Scientific Inc) Fluorometer. 150 bp paired-end reads were sequenced on Illumina HiseqX (Illumnia Inc.). Raw reads were quality checked with the FASTQC package. Adapters were removed as required for each kit using the cutadapt software and tolerating 10% error. Raw FASTQ files for each sample were uploaded onto the Partekflow suite for downstream analysis. Pre-alignment quality assurance/quality control (QA/QC) was performed, and reads were trimmed at the 3-prime (right end) and aligned to the mouse genome using STAR. The aligned reads were counted and annotated against the latest USC mouse transcript set on Partek to determine gene expression levels and counts normalized using the TMM (Trimmed Mean of M-value). Principal component analysis (PCA), hierarchical clustering and DEG analysis (p-value<0.05 and FC>1.5 or <-1.5) were all performed on Partek. DEG were identified using either ANOVA or the Gene Specific Analysis function (GSA, a Partek statistical modeling approach to test for differential expression of transcripts). Heatmap shows log2 fold changes calculated relative to the first condition listed in each comparison: 5mAS vs WT, and EV-treated 5mAS vs non-treated 5mAS. Color intensity reflects the magnitude of differential expression relative to the indicated reference condition.

10x Genomics Spatial Transcriptomics analysis of mouse kidneys.

We performed Visium 10x Genomics on 4 different samples: a healthy WT mouse (WT), 2m old AS, 5m old AS, and AS mice treated with one injection of hAFSC-EVs at 2m old and sacrificed 5m old. Samples were embedded in Tissue - Tek O.C.T. compound (Sakura Finetek USA, C #4583) and cryostored and sectioned to 10 μm thickness (Leica, Rotary Cryostat CM1510). RNA was isolated using the Qiagen RNeasy Mini kit (Qiagen, C#74104), and RNA quality was assessed using the 2100 Bioanalyzer (Agilent Technologies). All tissues had an RNA integrity number (RIN) above 9. Tissues were then sectioned onto pre-equilibrated Visium tissue optimization or gene expression slides (10x Genomics). These sections were then fixed in chilled methanol, stained with hematoxylin and eosin, and then imaged with an Aperio AT Turbo (Leica Biosystems) at 20X magnification. Automated real - time stitching of tiled images yielded a final image of the whole slide, which was imported to image analysis software (ImageScope, Leica Bio Systems). Using Visium Spatial Gene Expression Reagent Kits–Tissue Optimization User Guide (10x Genomics), tissues on optimization slides were permeabilized in a time course experiment and reverse transcription performed using fluorescently labeled nucleotides, resulting in fluorescent cDNA bound to the capture areas. Tissues were enzymatically removed, and fluorescence imaging was performed using a Zeiss Axio Scan.Z1 (Carl Zeiss Microscopy) digital slide scanner with a Texas Red filter set. Whole slide scanning was carried out at 20X with 150 ms exposure per image frame. Sequentially imaged frames were automatically stitched by the data acquisition software (Zen 3.0). After H&E staining and imaging of tissue sections on gene expression slides, the sections were permeabilized for 18 minutes to release poly - adenylated mRNA from overlying cells onto the capture areas of the slide. A permeabilization time of 18 min resulted in the maximum fluorescence signal with the lowest signal diffusion. Following the manufacturer's user guide (10x Genomics), the bound mRNA was then reverse transcribed, resulting in spatially barcoded, full-length cDNA. Second-strand synthesis was performed, followed by denaturation and transfer of the cDNA from the slide to a PCR tube. qPCR (KAPA

SYBR FAST qPCR Master Mix, Roche Sequencing and Life Science, KAPA Biosystems, Wilmington) was used to determine the number of cDNA amplification cycles required. After cDNA amplification, fragment analysis of the cDNA was performed on a 4200 Tape Station (Agilent Technologies). Library construction, performed on a portion of the cDNA, consisted of enzymatic fragmentation of the cDNA, end repair, and A-tailing, followed by double - sided size selection. Libraries were sequenced on a NovaSeq 6000 using a custom paired - end sequencing protocol, consisting of: read 1, 28 cycles; index 1, 10 cycles; index 2, 10 cycles; read 2, 90 cycles. The four samples (WT, 2m AS, 5m AS, and treated AS) were sequenced at the sequencing depths of 456M, 406M, 400M, and 412M reads and detected 4017, 3060, 2527, and 2865 spots in the samples, respectively. This corresponded to a median of unique molecular indices (UMIs) per spot of 19538, 8472, 14939, and 8818, and a median genes per spot of 4555, 3478, 4243, and 3406.

Raw sequencing data were demultiplexed and converted to FASTQ format by using bcl2fastq v2.20. Space Ranger software v1.0.0 (10x Genomics) was used for read alignment, tissue and fiducial frame detection, and barcode/UMI counting with predefined default parameters. Briefly, raw reads were aligned to the rat reference genome (Rnor6.0), collapsed by UMI, and counted.

The samples were integrated together with Seurat 71 v4.0.5 in R v4.1.1 using the functions SCTransform followed by FindIntegrationAnchors. To obtain two-dimensional projections' dynamics, principal component analysis (PCA) was run on the SCTransformed gene - barcode matrix to reduce the number of feature dimensions. After running PCA, a UMAP algorithm was applied on the top 20 principal components to further reduce these components to visualize cells in a two-dimensional space. Based on their relative positions in the UMAP plot, an unsupervised graph - based clustering was performed to group cells for the clustering analysis. To identify molecular features that correlate with spatial location within a tissue, differential expression is performed based on the normalized RNA expression matrix using pre - annotated anatomical regions or Louvian clusters with the Seurat function FindMarkers. The manual annotation was based on known renal cell-type markers (8) and was consistent with Seurat and Harmony

generated annotations. Data from each sample were visualized for exploration via Loupe Cell Browser. Significant DEG (adj_p-value<0.05, log2FC>0.25 or log2FC<-0.25) were considered biologically relevant and used for downstream GO analysis (https//:geneontology.org; https://rgd.mcw.edu).

CosMx Spatial Molecular Imager Sample Preparation and Data Analysis.

Sample Preparation: A 5µm section from a human kidney sample was profiled using the CosMx spatial molecular imager (9). In brief, the samples were mounted on a histological slide, and a flow cell for reagent administration was affixed to the slide. Once the flow cell was loaded onto the instrument, the entire flow cell was scanned, and 29 fields of view (FOVs, 0.9mm x 0.7mm) were placed on each slide for RNA readout. The FOVs (covering the entire biopsy tissue) were stained for a cytoplasmic membrane cocktail (blue channel), PanCK (green), CD45 (yellow), CD3 (red), DAPI (UV), and profiled with the 1000-plex Universal Cell Characterization panel. Images of the targets on each slide were captured, which were subsequently decoded based on their assigned barcode sequences and localization. Study limitation: Biopsy tissue was not available for RIN or DV200 analysis to evaluate RNA quality, representing a limitation in our study design.

Cell Segmentation and Assignment of Transcripts: Segmentation was performed using a consensus model of the DAPI channel alone and the composite projection of all other stains to draw cell boundaries on the samples. A cell segmentation pipeline using a machine learning algorithm (10-11) was used to accurately assign transcripts to cell locations and subcellular compartments. The transcript profile of individual cells was generated by combining target transcript location and cell segmentation boundaries. Cells with fewer than 20 total transcripts assigned were omitted from analysis. Analysis was performed using R package Seurat v4.0.5 in R v4.1.1.

Cell Type Annotation: Cell type classification was performed using the InSituType package (12). An atlas of healthy and injured kidney cells (13) and single-cell RNA-seq of the Adult Human

Kidney (Version 1.0) file from https://cellxgene.cziscience.com were used as a reference dataset for supervised cell typing. Some cell types were merged via the InSituType::refineClusters function to improve confidence of calls. After merging, cells with an accurate cell classification less probable than .80 percent were removed from analysis. Images showing probe glomerular localization were processed using Napari 0.4.17 imaging software (14). For the Volcano plot, DEG with log2FC>0.2 or log2FC<-0.2; adj_p-value<0.05 were considered.

References for Supplementary Methods

1. Sedrakyan S, et al. Amniotic fluid stem cell-derived vesicles protect from VEGF-induced endothelial damage. *Scientific Reports*. 2017;7(1):16875.
2. Perin L, et al. Protective effect of human amniotic fluid stem cells in an immunodeficient mouse model of acute tubular necrosis. *PLoS One*. 2010;5(2):e9357.
3. Carraro G, et al. Human amniotic fluid stem cells can integrate and differentiate into epithelial lung lineages. *Stem Cells*. 2008;26(11):2902–11.
4. Garcia O, et al. Amniotic fluid stem cells inhibit the progression of bleomycin-induced pulmonary fibrosis via CCL2 modulation in bronchoalveolar lavage. *Plos One*. 2013;8(8):e71679.
5. Petrosyan A, et al. A glomerulus-on-a-chip to recapitulate the human glomerular filtration barrier. *Nature Communications*. 2019;10(1):3656.
6. Zhang Q, et al. C3aR-initiated signaling is a critical mechanism of podocyte injury in membranous nephropathy. *JCI Insight*. 2024;9(4):e172976.

7. Huang DW, et al. The DAVID Gene Functional Classification Tool: a novel biological module-centric algorithm to functionally analyze large gene lists. *Genome Biol*. 2007;8(9):R183.
8. Novella-Rausell C, et al. A comprehensive mouse kidney atlas enables rare cell population characterization and robust marker discovery*. iScience*. 2023;26(6):106877.
9. He S, et al. High-plex imaging of RNA and proteins at subcellular resolution in fixed tissue by spatial molecular imaging. *Nat Biotechnol*. 2022;40(12):1794-1806.
10. Stringer C, et al. Cellpose: a generalist algorithm for cellular segmentation. *Nature methods*. 2021;18(1): 100-106.
11. Pachitariu M, Stringer C. Cellpose 2.0: how to train your own model. *Nature methods*. 2022;19(12):1634-1641.
12. Danaher P, et al. Insitutype: likelihood-based cell typing for single cell spatial transcriptomics. *bioRxiv.* 2022;10.19.512902.
13. Lake BB, et al. An atlas of healthy and injured cell states and niches in the human kidney. *Nature*. 2023;619(7970):585-594.
14. Ahlers J, et al. napari: a multi-dimensional image viewer for Python. *Zenodo*. 2023;(v0.4.18).
