# Supplementary material for: Extracellular vesicle miR-93-5p cargo regulates glomerular endothelial cell damage in Alport syndrome
Source: JCI Insight. 2026 Mar 23;11(6):e197643. doi: 10.1172/jci.insight.197643 (PMC13043101; doi:10.1172/jci.insight.197643)

Original WB for all Figures

Extracellular vesicle-miR-93-5p cargo regulates glomerular endothelial cell damage in Alport syndrome.

Charmi Dedhia, Valentina Villani, Xiaogang Hou, Paolo Neviani, Geremy Clair, Mohammadreza Kasravi, Cristina Grange, Paolo Cravedi, Paola Aguiari, Velia Alcala, Giuseppe Orlando, Xue-Ying Song, Johnathan E Zuckerman, Roger E De Filippo, Stefano Da Sacco, Sargis Sedrakyan, Benedetta Bussolati, Laura Perin

Figure 3B

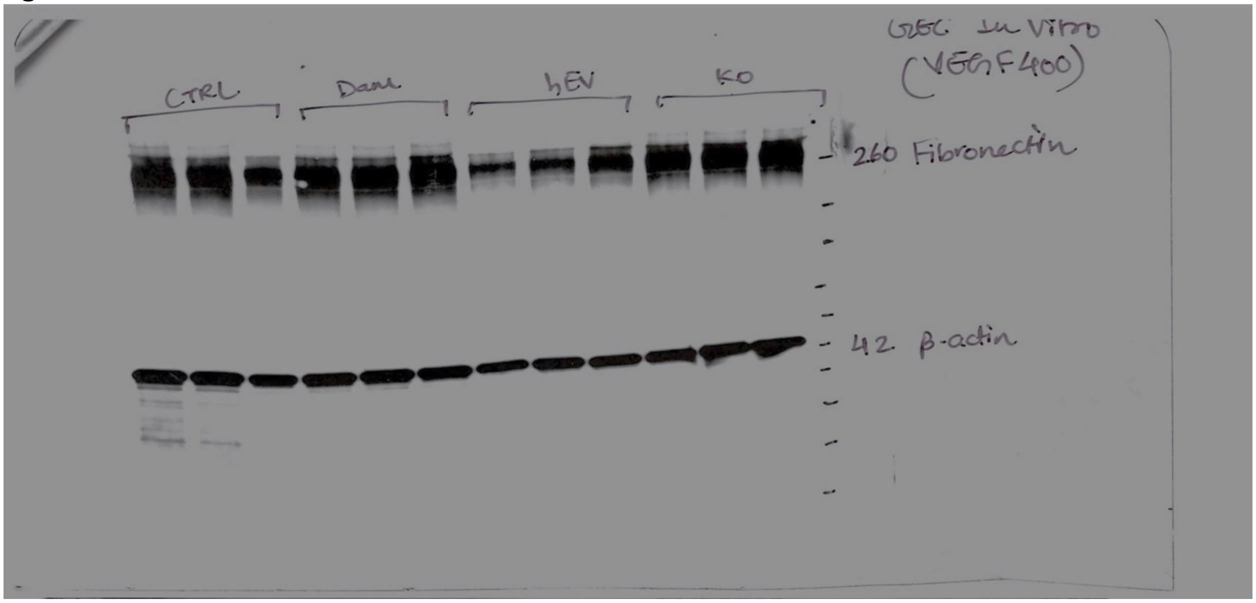

Figure 3D

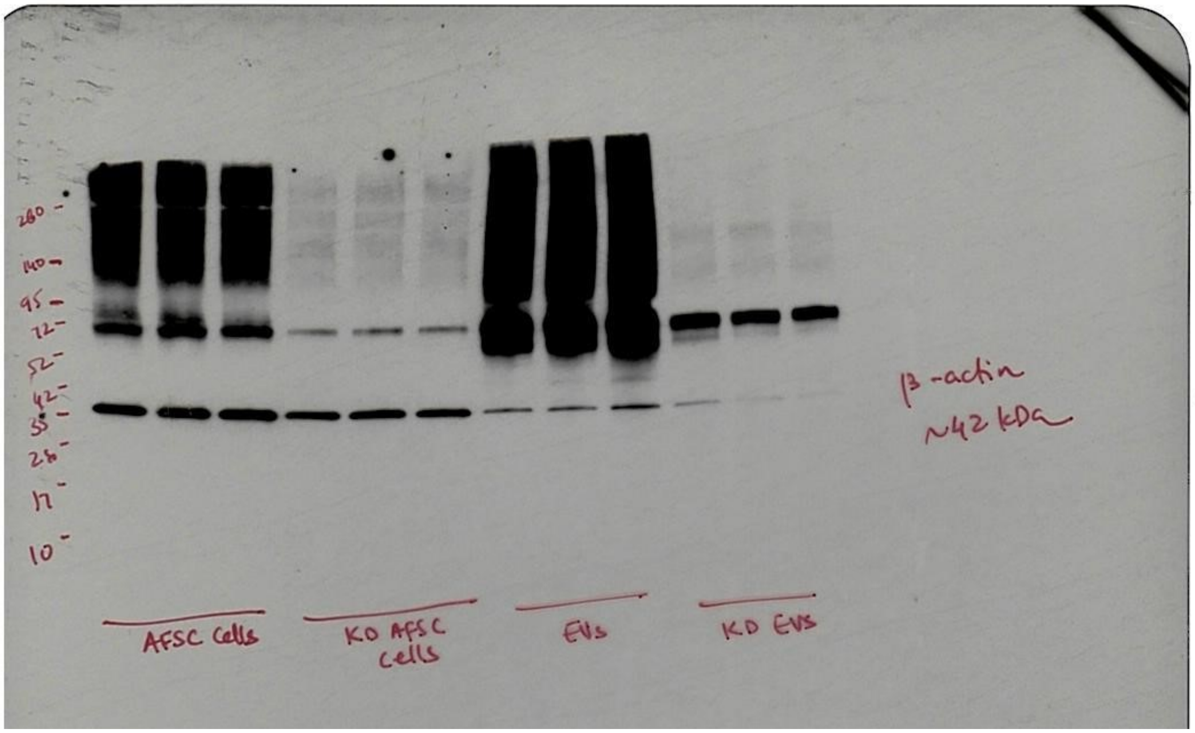
260
140
95
72
52
42
35
26
17
10
AFSC Cells
KO AFSC cells
EVs
KD EVs
β-actin
~42 kDa

Figure 3E

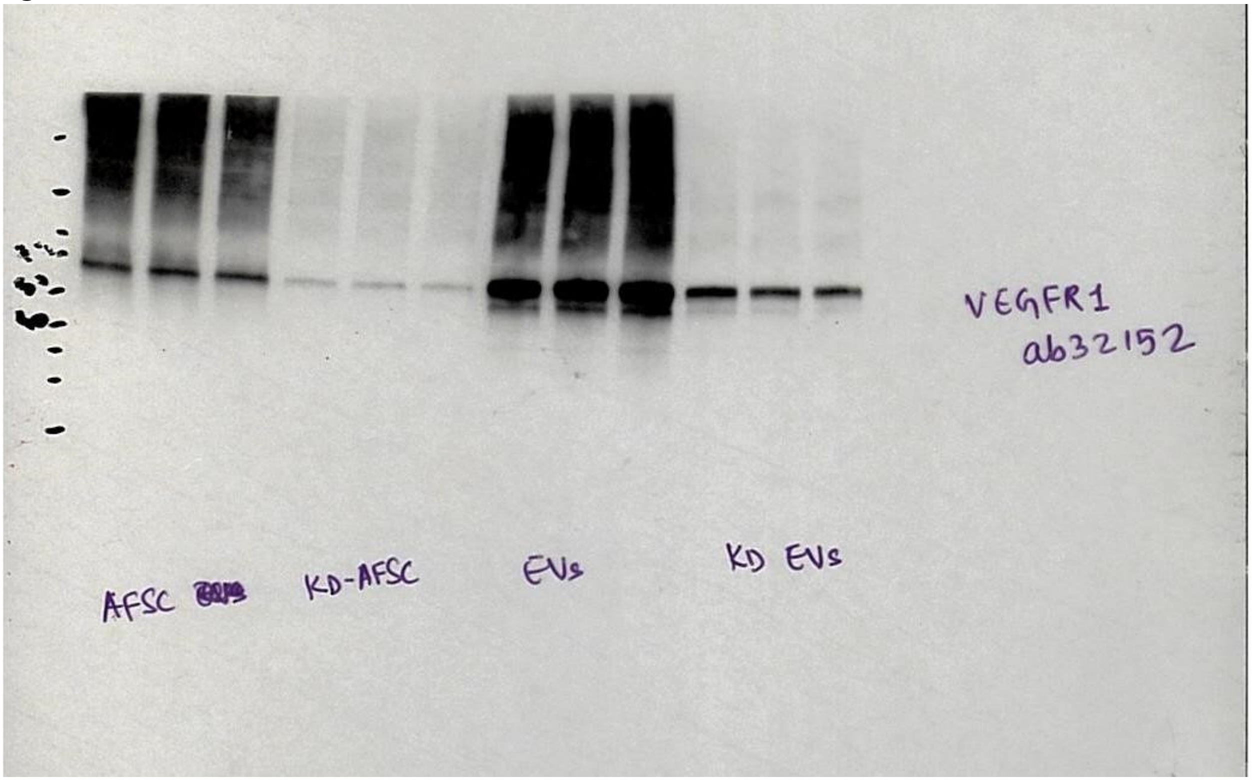

Figure 3F

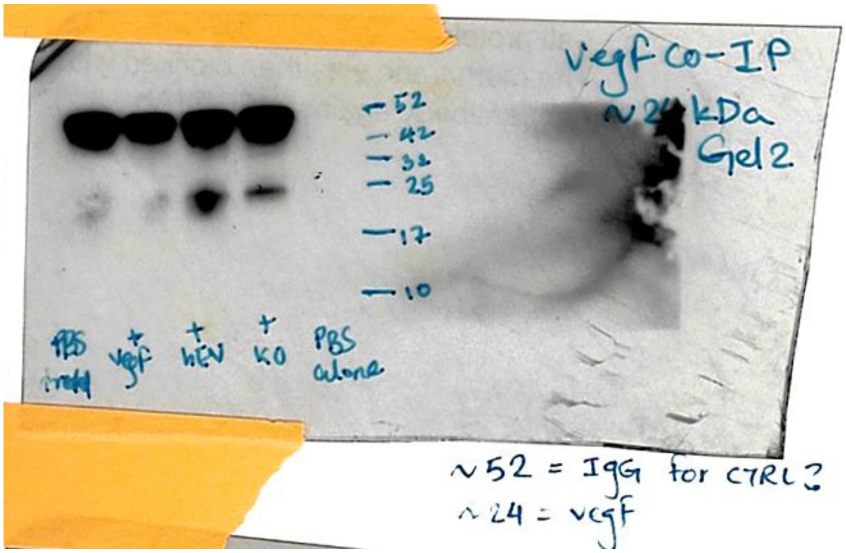

Figure 3G

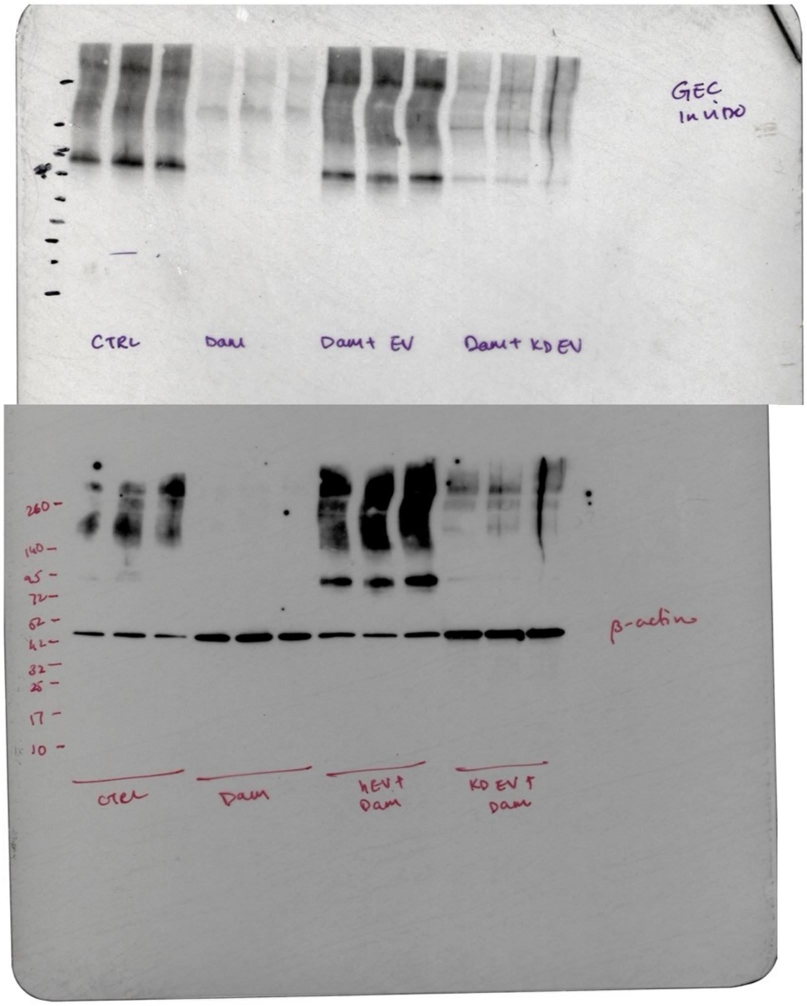
GEC
CTRL
Dam
Dam+ EV
Dam+ KD EV
260
140
95
72
52
42
32
25
17
10
β-actin
ctrl
Dam
hEV+ Dam
KD EV + Dam

Figure 3H

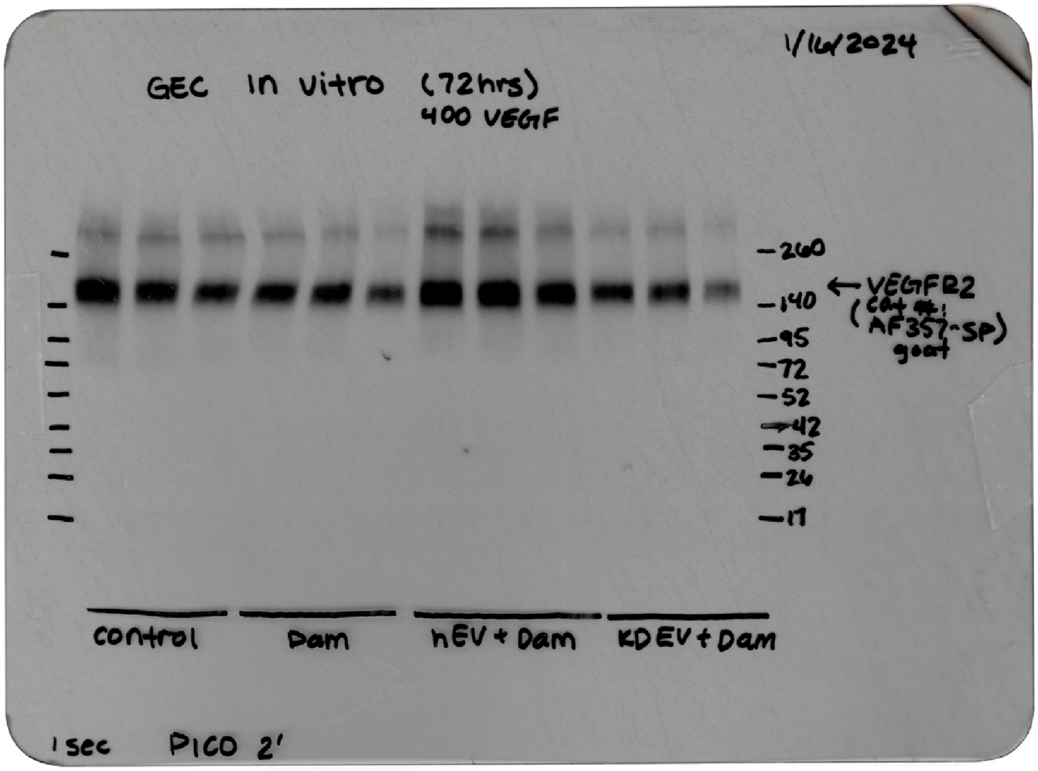
1/16/2024
GEC in vitro (72hrs)
400 VEGF
260
140
95
72
52
42
35
26
17
← VEGFR2
(Cat #: AF357-SP)
goat
control
Dam
hEV + Dam
KDEV + Dam
1 sec
PICO 2'

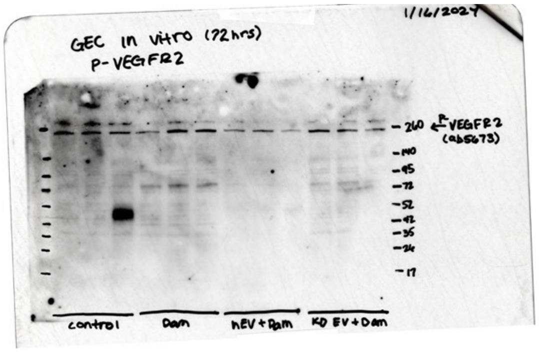
1/16/2024
GEC in vitro (72hrs)
P-VEGFR2
260
VEGFR2
control
Dam
hEV + Dam
KD EV + Dam

Figure 5H

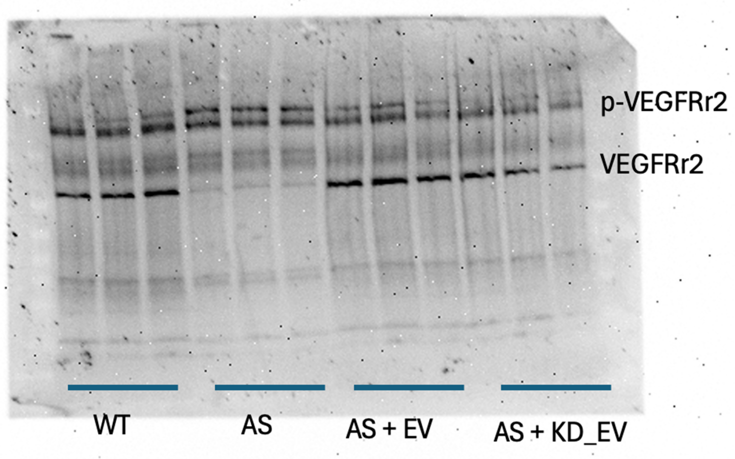

Figure 5I

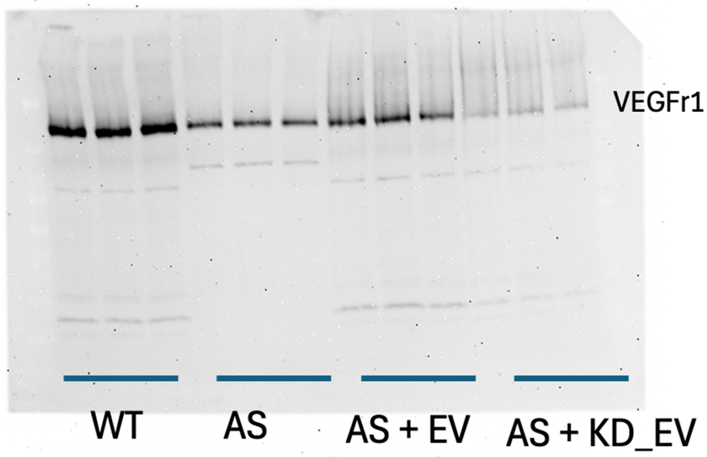

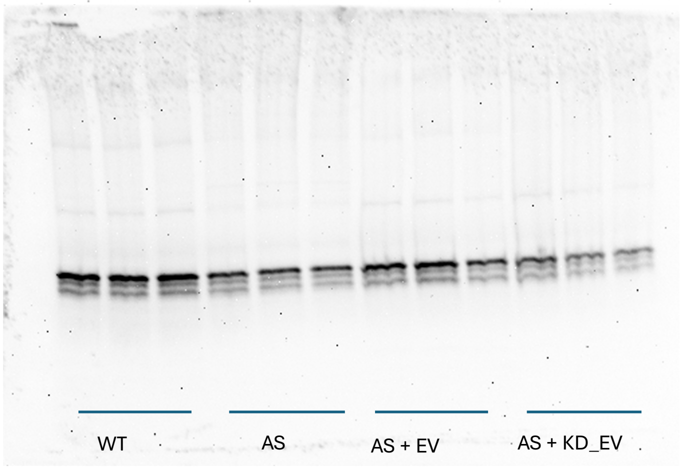

Suppl. Figure 6B

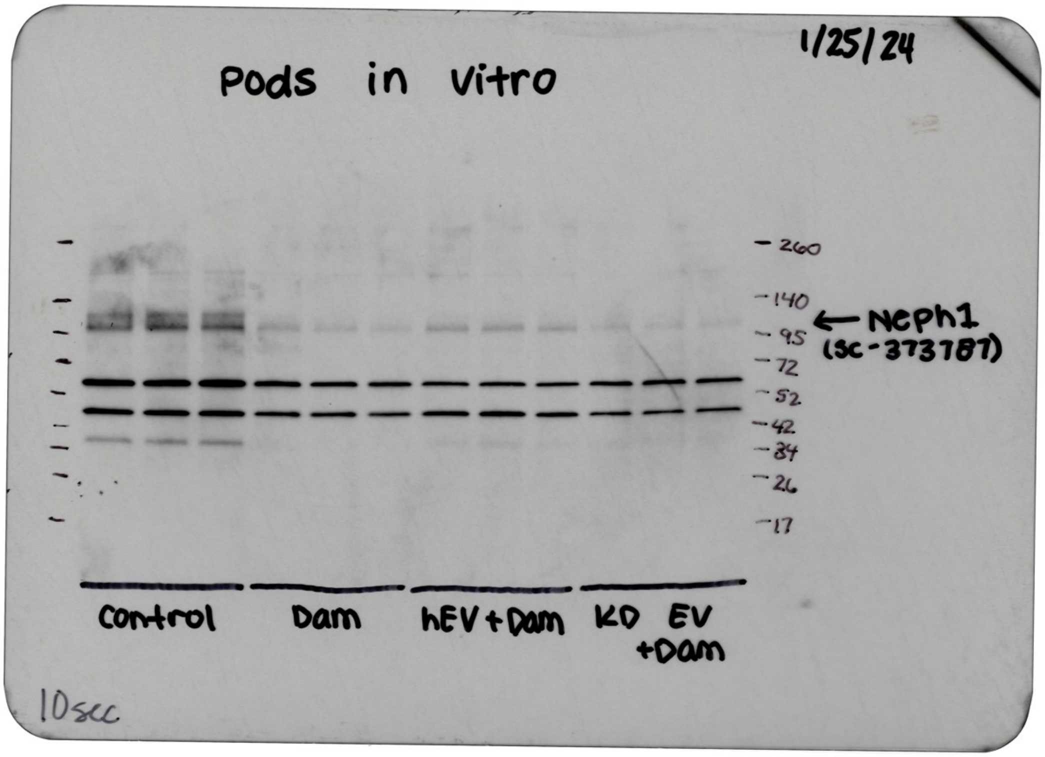

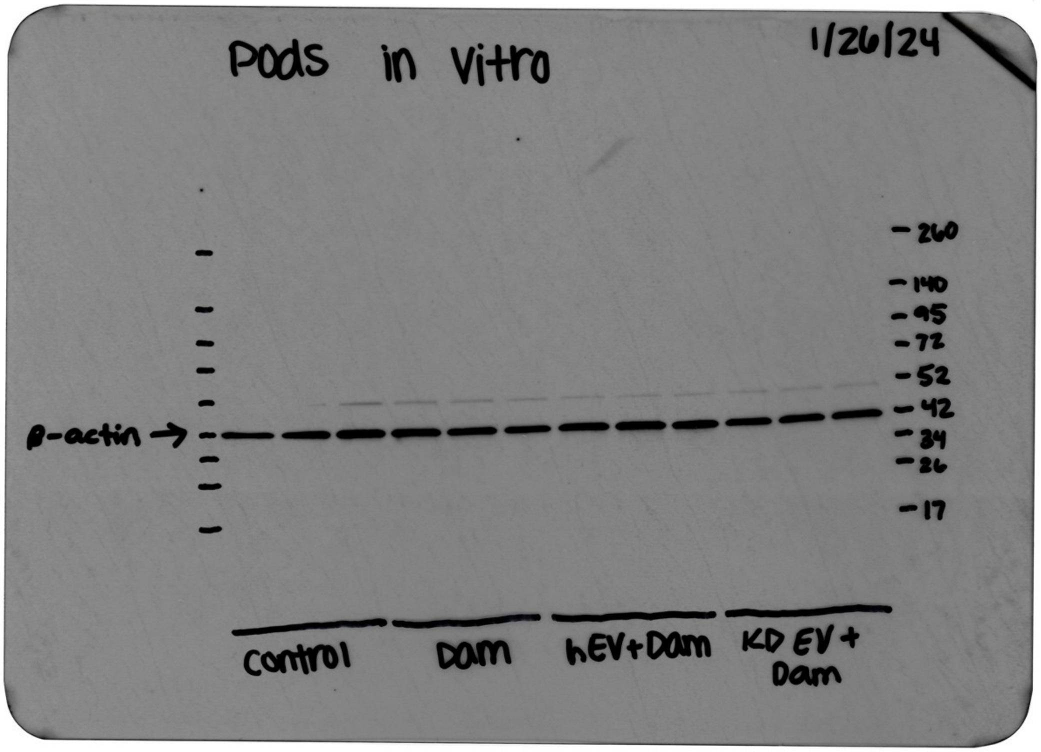

Suppl. Figure 6D

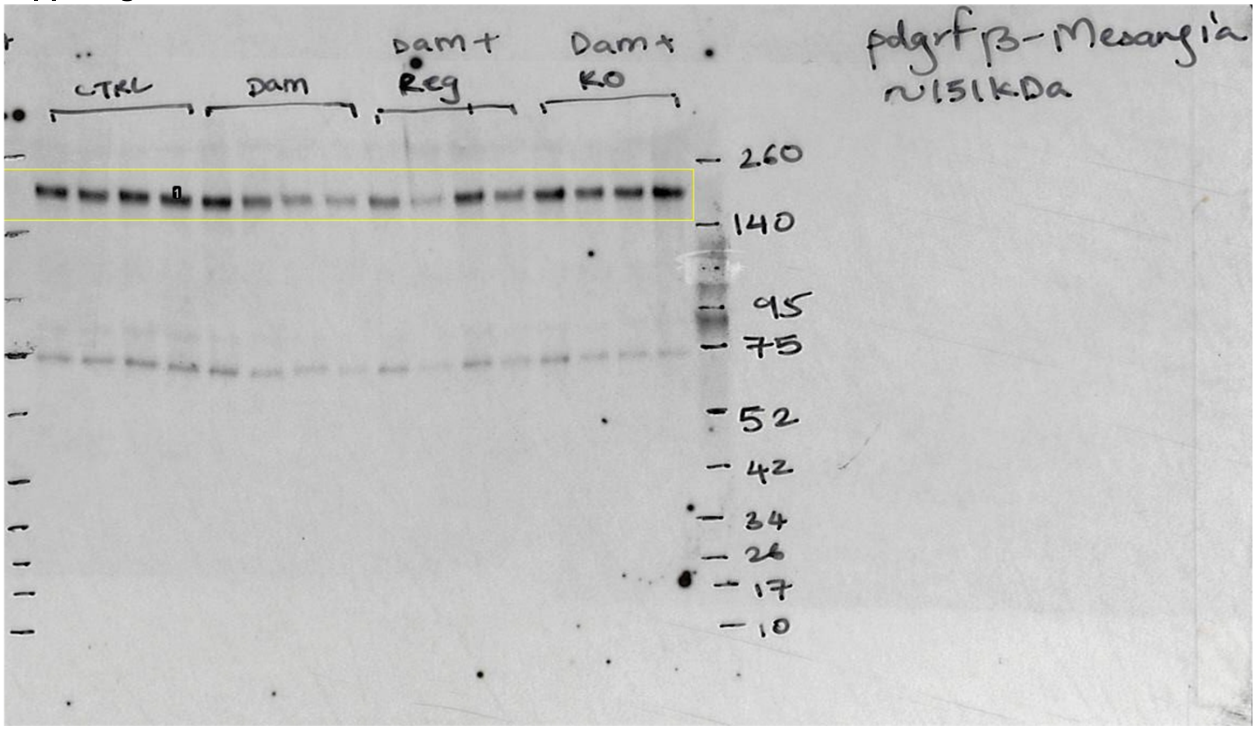

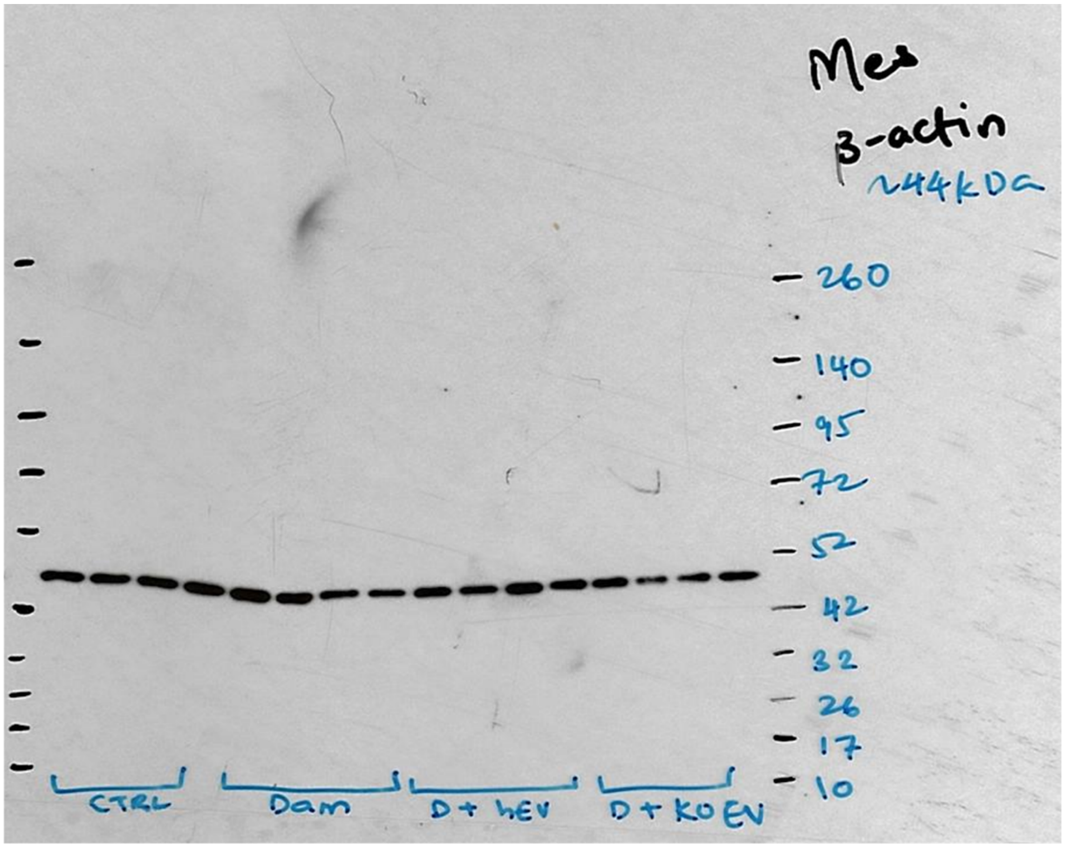

Supplement: Unedited blot and gel images [file jciinsight-11-197643-s164.pdf]
